# Supplementary material for: Specialized rainforest hunting by Homo sapiens ~45,000 years ago
Source: Nat Commun. 2019 Feb 19;10:739. doi: 10.1038/s41467-019-08623-1 (PMC6381157; doi:10.1038/s41467-019-08623-1)
Supplement: Supplementary file 1 — Supplementary Information [file 41467_2019_8623_MOESM1_ESM.pdf]

Supplementary Information

Specialized rainforest hunting by *Homo sapiens* ~ 45,000 years ago

Wedage et al.

This PDF file includes:

Supplementary Note 1. Further notes on stratigraphic phasing
Supplementary Note 2. Further detail relating to faunal analyses
Supplementary Note 3. Lithic Materials
Supplementary Figures 1-19
Supplementary Tables 1-44
Supplementary References

Supplementary Note 1. Further notes on stratigraphic phasing

The sediment fill of Fa-Hien Lena consists of ca. 170 cm of detrital sediment, representing at least 4 four major phases of occupation (Supplementary Figure 1) deposited above heavily weathered and karstified gneiss blocks, (phantomed bedrock and/or roof fall blocks) and steeply dipping, folded gneiss (Supplementary Figures 2-3).

Phase D: Late Pleistocene Occupation

Sediments directly above the gneiss slabs and bedrock comprise pebbly loams with angular spalls and sand, resulting from the disintegration of gneiss blocks, and clayey deposits which might have resulted from colluviation of soils/saprolith from outside. These sedimentary layers yielded faunal remains (28.7% of which showed evidence for burning and calcination), shell beads, ochre fragments, bone points, and quartz flakes, suggesting human presence in the cave. Above these facies is a ca. 30-50 cm thick succession of sharp-based, sub-horizontal layers of dark, sandy silt and laminated ash deposits that drape and interfinger with at least two distinct accumulations of angular gneiss blocks. These deposits are rich in charcoal and other organics and contain numerous faunal remains and artifacts.

Micromorphological analysis of four samples from Phase D deposits (corresponding to contexts 92, 91, 90; contexts 89, 88, 87; context 62 in the 2009 profile, Supplementary Figure 2) reveals various coarse grained microfacies with abundant and remarkably well preserved human occupation debris. Basal Phase D deposits consist of cm-scale beds and laminae of *in situ* phospathised ash intercalated with gneiss pebbles and microaggregated organic loam with abundant charcoal, exceptionally well preserved uncharred plant remains and phytoliths (Supplementary Figure 4). Many of the plant remains are probably palm fronds (identified by the presence of articulated phytoliths). Larger calcitic particles within the ash deposits may be burned snail shells. Angular microflakes or clear quartz probably originated as knapping residue. Alongside bioturbation by mesofauna, which accounts for the crumb microstructure of loamy deposits, the most striking postdepositional changes of these early occupation deposits are diagenetic: pervasive phosphatisation of ash, dissolution of bone and ash, and growth of xenotropic gypsum nodules that disrupt the primary lamination of ash deposits. These diagenetic products, similar to those reported from guano-rich deposits of other tropical[1, 2] and temperate[3, 4] caves, suggest that bat guano was deposited in Fa-Hien Lena, perhaps between episodes of human occupation.

Stratigraphically higher Phase D deposits contain layers of structureless, pebbly ash with angular gneiss clasts and numerous burning residues (wood charcoal, burned bone, snail shell, abundant phyoliths and burned sediment intraclasts, interpreted as rip up clasts) floating in a matrix of rhombic calcite crystals (derived from wood ash). These are interpreted as midden deposits, resulting from the dumping and/or colluvial reworking of human occupation debris – predominantly hearth and cooking residues – into the back of the cave. Upper Phase D deposits, immediately under the large gneiss slabs (correlative with context 157(?) in the 2010 excavation: Supplementary Figure 2) are matrix-supported organic breccia with abundant burned and unburned biogenic residues (including well preserved fragments of *Canarium* sp. shells) in a

matrix of silty clay and (local) accumulations of calcite rhombs (reworked wood ash). Imbrication of gneiss pebbles/granules and other platy particles suggests deposition from flow – probably surface wash – directed towards the cave interior. These deposits are also interpreted as colluvia originating in middens or other habitation floor deposits.

Phase D sediments, therefore, record intermittent/episodic human occupation, from around 48,000 – 33,000 cal. BP (MIS 3) and colluvial inwash, during a period of structural instability of cave walls and ceiling (which accounted for the roof fall blocks embedded within the fine-grained facies of this phase). The latest of these roof fall episodes appears to have contributed to the exceptional preservation of these deposits by sealing large parts of Phase D from later disturbance. Human-mediated deposits evidence hearth building and maintenance, cooking, consumption and discard of food remains (which included *Canarium* sp. and other, as yet unidentified plant remains), and, possibly, the use of palm fronds for the construction of artefacts (e.g., mats, sleeping platforms, baskets and similar containers, as inferred from other Pleistocene sites in Sri Lanka: e.g., Batadomba-lena[3]. The depositional and diagenetic history these sediments will be reported elsewhere.

Phase C: Terminal Pleistocene Occupation

Phase C sediments are a very heterogeneous mixture of dark colored (dark gray to brown), organics-rich sandy silty loams, sandy loams and matrix-supported breccia with abundant charcoal and ash. Ash and charcoal are mixed with detrital sediments and also present as distinct laminae or lenses. Phase C deposits are structured as sharp-based layers inclined towards the cave wall, or as fills of 10s-of-cm-sized pits. With the exception of charcoal and ash laminae, bedding is absent; clast-rich units are often structureless, with gneiss clasts positioned at high angle. Despite its relatively narrow temporal range (13,000-12,000 cal. BP), Phase C contains the densest concentration of artifacts and human occupation debris in the entire Fa-Hien stratigraphy.

Micromorphological analysis of one sample from basal Phase C deposits (near, or perhaps across the Phase D/C boundary: Supplementary Figure 1) shows a very sharp based accumulation of closely packed wood charcoal and other charred biogenic particles (other plant remains, bone, snail shell) in a matrix of fine grained charred organic debris. Elongate particles are imbricated and register parallel bedding. This deposit may have resulted from relatively high-energy floor wash (e.g. a coarse lag formed in a floor depression) or from dumping or raked hearth residues. Calcitic ash, conspicuously absent from these burning residues, may have dissolved postdepositionally.

Truncation of stratigraphic boundaries, juxtaposed cut-and-fill features, absence of intrastratal structure and the overall mixed aspect of its deposits suggest that Phase C resulted from a succession of erosion, colluviation and, perhaps, dumping episodes. It is difficult to work out the relative chronology of these episodes based on current evidence. Floor hollows were excavated in floor wash colluvia (perhaps also including mass flow deposits) rich in reworked human occupation debris, and other occupation debris-rich deposits dumped or deposited *in situ* at the rear of the cave.

Colluviation and dumping of excavated debris could have resulted in mixing of charcoal and other datable sediment components of different ages; yet such mixing is not apparent in the

current set of dates. The clustering of radiocarbon dates around 13,000 to 12,000 cal. BP. may indicate rapid deposition.

Phase B: Early Holocene Occupation

Phase B is made up of two distinct lithostratigraphic units separated from the Terminal Pleistocene deposits by a sharp boundary that truncates underlying strata. The first unit consists of a series of subhorizontal layers of light brown sandy silts, matrix-supported collapse breccia and ash accumulations, with a moderate amount of artifacts and habitation debris, interpreted as undisturbed occupation deposits and floor wash colluvia (in contrast to the dumped and reworked deposits of Phase C). The second unit includes a large (diameter: 85 cm, depth: 80 cm), multi-stage pit and its fill. The pit fill, dated to ca. 8000-7700 cal. BP., consists of multiple layers of sandy silt and steeply-dipping accumulations of ash with abundant charcoal, much of which is charred *Canarium* sp. endocarps. Fire-reddening of the immediate substratum of some ash deposits suggests that burning may have taken place *in situ*; structureless mixtures of ash and charcoal may have resulted from deliberate ash dumping. Other fill units may have been deposited through floor wash and related colluvial processes.

Phase A: Mid-Holocene Occupation

Like Phase B, Phase A also comprises two distinct stratigraphic units. The first unit is composed of brown sandy loams and lenses of *Canarium* endocarp charcoal deposited directly above the pit. These deposits appear to have resulted from the building of hearths *in situ* (the thick accumulation of charred *Canarium* endocarp may represent several burning episodes on the same spot) followed by colluvial deposition on the cave floor. This unit is dated to ca. 5900 cal. BP. Above these deposits come ca. 25-35 cm of sharp-based, brown sandy and silty clays with little internal structure. These deposits are interpreted as dumps, derived from prehistoric habitation contexts. Dates of ca. 4700-4500 cal. BP. have been published[4]. Overlying the Mid-Holocene deposits is a thick brown sandy silt layer with high density of spalls followed by subhorizontal layers of sandy silty loams immediately under the present cave floor. These deposits resulted from extensive mining of the cave floor for fertilizer in the recent period, and even more recent colluvial deposition on the cave floor.

Supplementary Table 1. Radiocarbon dates from the 1986 excavations[5].

Sample*	Context	Lab. Code	Measured
B-N5-2	2	Beta-33297	4,750 ± 60
B-M6-2	3	Beta-33293	6,850 ± 80
B-N6-2a	3a	Beta-33298	7,100 ± 60
B-M7-3	4	Beta-33295	24,470 ± 290
B-N7-3	4	Beta-33299	30,060 ± 290
B-M7-5	4a	Beta-33296	32,060 ± 630
B-M6-6	5	Beta-33294	33,070 ± 630

*Bulk radiocarbon measurement made on charcoal.

Supplementary Table 2. Radiocarbon dates from the 2010 excavations.

Sample†	Context	Measured	Conventional
BYP2010/CX NE/N-4, O-4, 107	107	3910 ± 30	3870 ± 30
BYP 2010 CX NE/0-6, 0-6, 116 middle	116	4,870 ± 40	4,800 ± 40
BYP2010/CX NE/N-4, O-4, 109	109	10,220 ± 40	10,150 ± 40
BYP 2010 CX NE/O-4, P-4, 108	108	33,260 ± 240	33,220 ± 240
BYP2010/CX NE/N-4, O-4, 110	110	36,950 ± 300	36,910 ± 300
BYP 2010 NE/O-4, 118	118	31,770 ± 190	31,750 ± 190
BYP 2010 CX NE/O-4, P-4, 119	119	10,300 ± 40	10,250 ± 40
BYP 2010 CX NE/ O-4, 126F	126	37,260 ± 310	37,230 ± 310

†AMS radiocarbon dates

Supplementary Table 3. Radiocarbon dates from the 2012 excavation.

Sample[†]	Context	Lab. Code	Measured	Conventional
135	31/32/135	Beta-354907	4,860 ± 30	4,820 ± 30
152	51/152	Beta-354916	7,030 ± 40	6,990 ± 40
152	51/152	Beta-355793	7,300 ± 40	7,240 ± 40
153	52/153	Beta-354917	7,040 ± 40	6,900 ± 40
136	136	Beta-354908	7,010 ± 30	6,970 ± 30
138	138	Beta-354909	7,750 ± 40	7,720 ± 40
237	237	Beta-354921	10,460 ± 40	10,390 ± 40
139	139/140	Beta-354910	10,390 ± 40	10,350 ± 40
141	141	Beta-354911	10,440 ± 40	10,340 ± 40
142	142	Beta-354912	10,500 ± 40	10,430 ± 40
174	174/246	Beta-354919	10,490 ± 40	10,440± 40
144	144/161/164	Beta-354913	10,330 ± 40	10,290 ± 40
145	145	Beta-354914	32,920 ± 240	32,890 ± 240
175	175	Beta-354920	34,610 ± 320	34,600 ± 320
159	159	Beta-354918	43,030 ± 720	43,000 ± 720

[†]AMS radiocarbon dates

Supplementary Table 4. Calibrated radiocarbon dates for Fa Hien-lena.

Sample	Context	Calibrated (cal. years BP)‡
BYP2010/CX NE/N-4, O-4, 107	107	4,422-4,248
B-N5-2	2	5,594-5,322
135	31/32/135	5,653-5,488
BYP 2010 CX NE/0-6, 0-6, 116 middle	116	5,710-5482
B-M6-2	3	7,916-7,570
136	136	7,935-7,762
152	51/152	7,954-7,763
153	52/153	7,955-7,791
B-N6-2a	3a	8,020-7,794
152	51/152	8,180-8,020
138	138	8,595-8,430
BYP2010/CX NE/N-4, O-4, 109	109	12,096-11,768
BYP 2010 CX NE/O-4, P-4, 119	119	12,380-11,844
144	144/161/164	12,386-11,910
139	139/140	12,419-12,062
141	141	12,530-12,120
237	237	12,549-12,131
174	174/246	12,575-12,150
142	142	12,590-12,236
B-M7-3	4	29,126-27,872
B-N7-3	4	34,656-33,686
BYP 2010 NE/O-4, 118	118	36,136-35,191
B-M7-5	4a	37,912-34,764
145	145	37,912-36,300
B-M6-6	5	38,826-35,828
BYP 2010 CX NE/O-4, P-4, 108	108	38,333-36,690
175	175	39,876-38,490
BYP2010/CX NE/N-4, O-4, 110	110	42,036-40,980
BYP 2010 CX NE/ O-4, 126F	126	42,228-41,258
159	159	48,046-45,028

‡All samples have been calibrated using the OxCal 4.3[6] software and IntCal calibration curve[7].

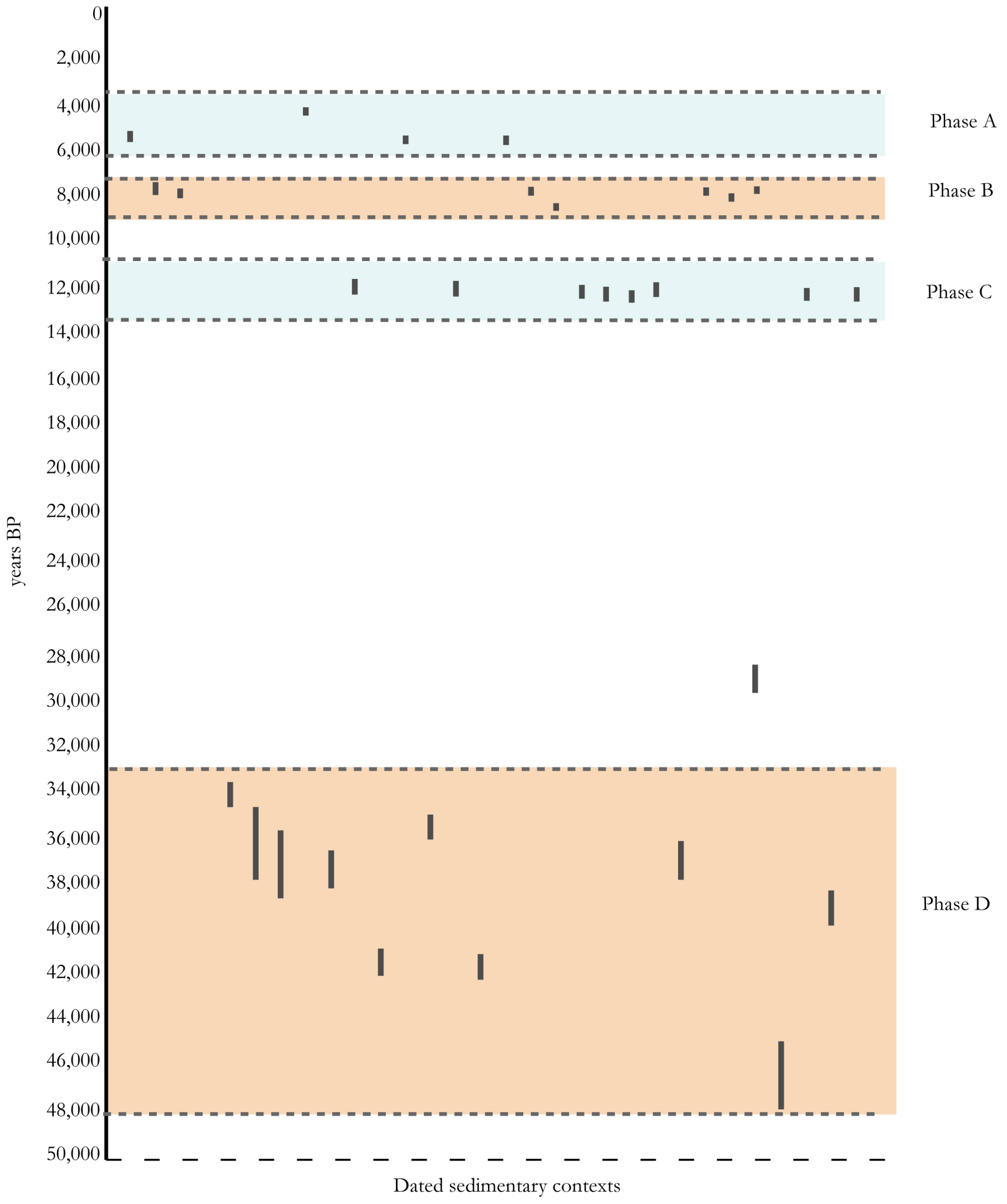

Supplementary Figure 1. Phases of human occupation in Fa-Hien Lena based on radiocarbon dates (cal. years BP). See Supplementary Table 4 for the list of dated sedimentary contexts.

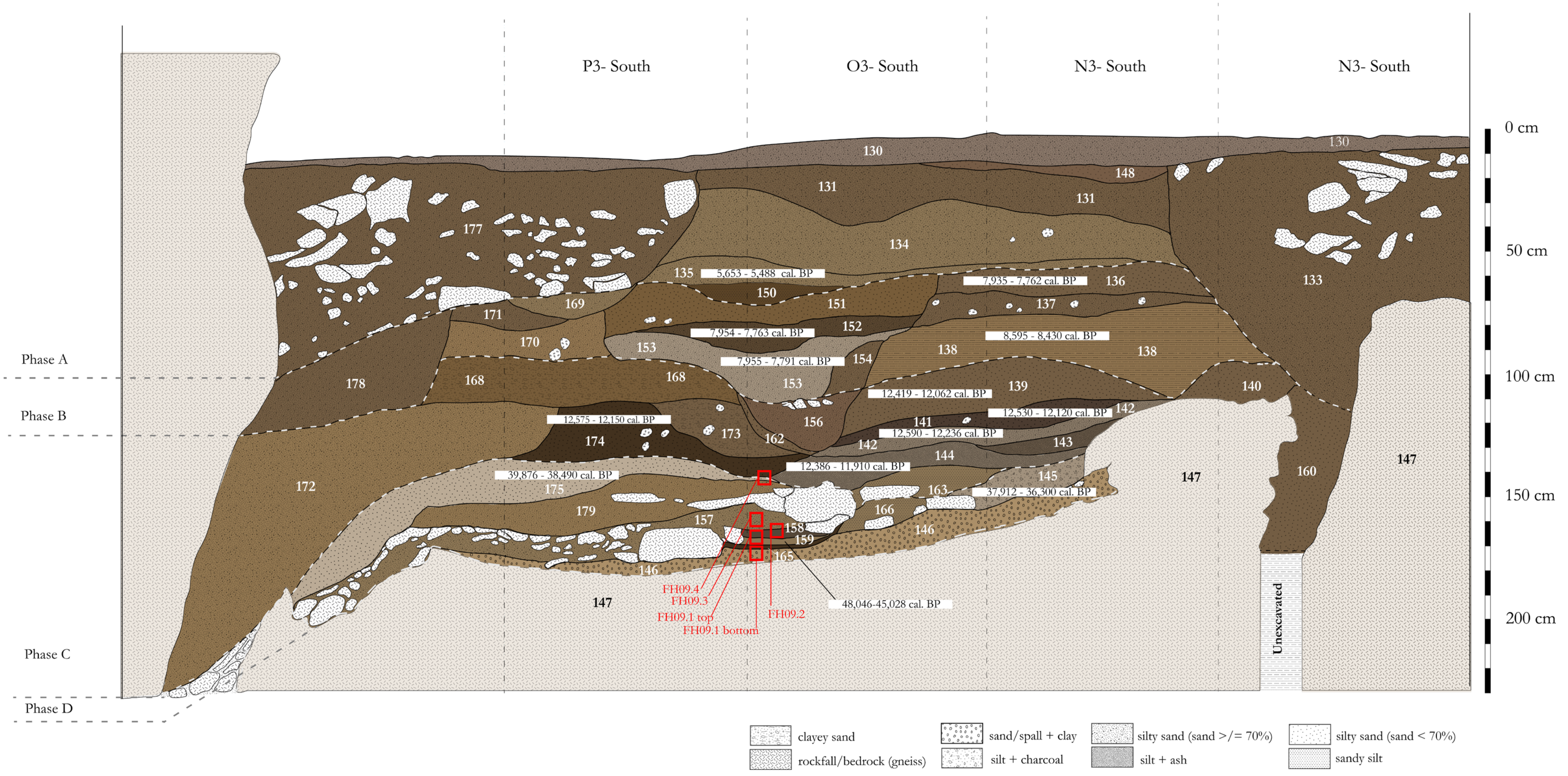

Supplementary Figure 2. Stratigraphy of Fa-Hien Lena, (south wall end of the 2010 excavation, colors represent Munsell color values of sediments). Frames: correlative stratigraphic position of thin section micromorphology samples collected in the 2009 excavation.

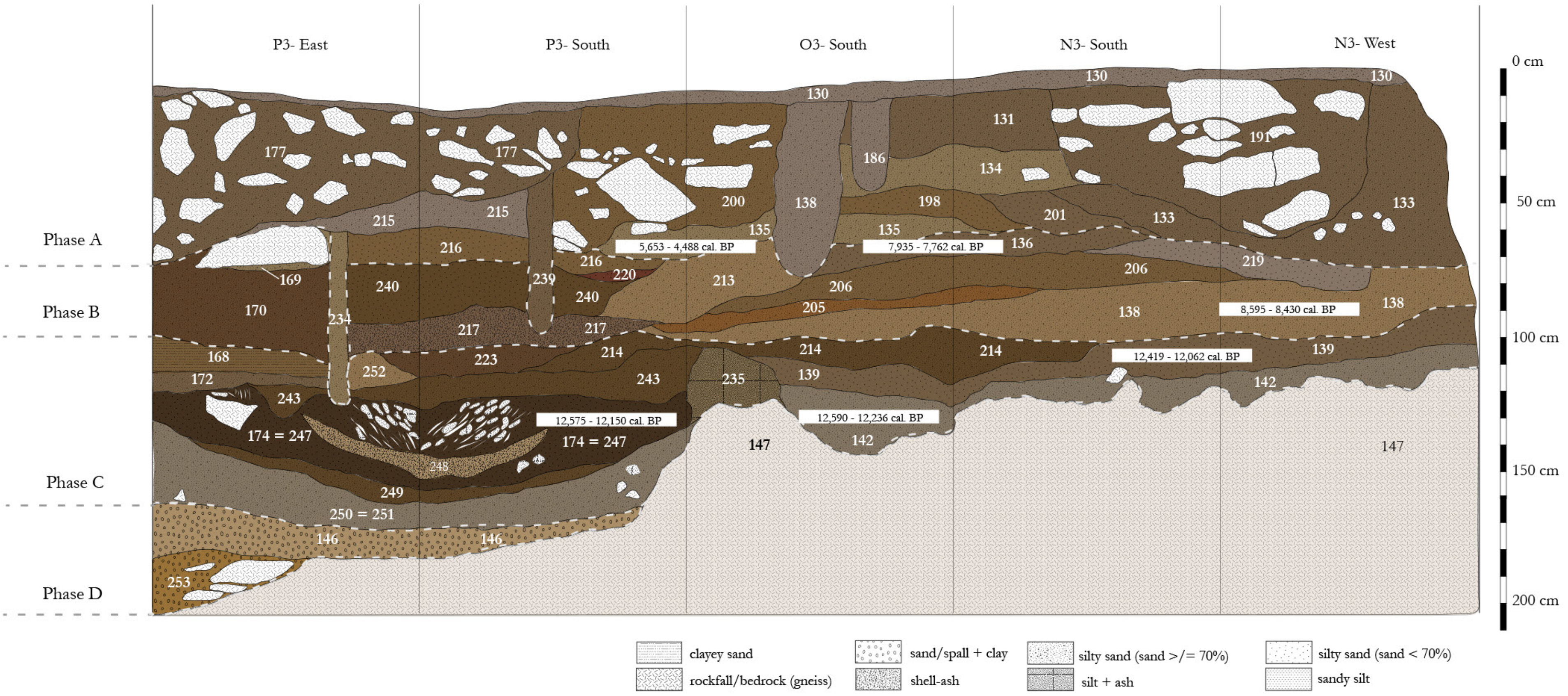

Supplementary Figure 3. Stratigraphy of Fa-Hien Lena (south wall end of the 2012 excavation, colors represent Munsell color values of sediments).

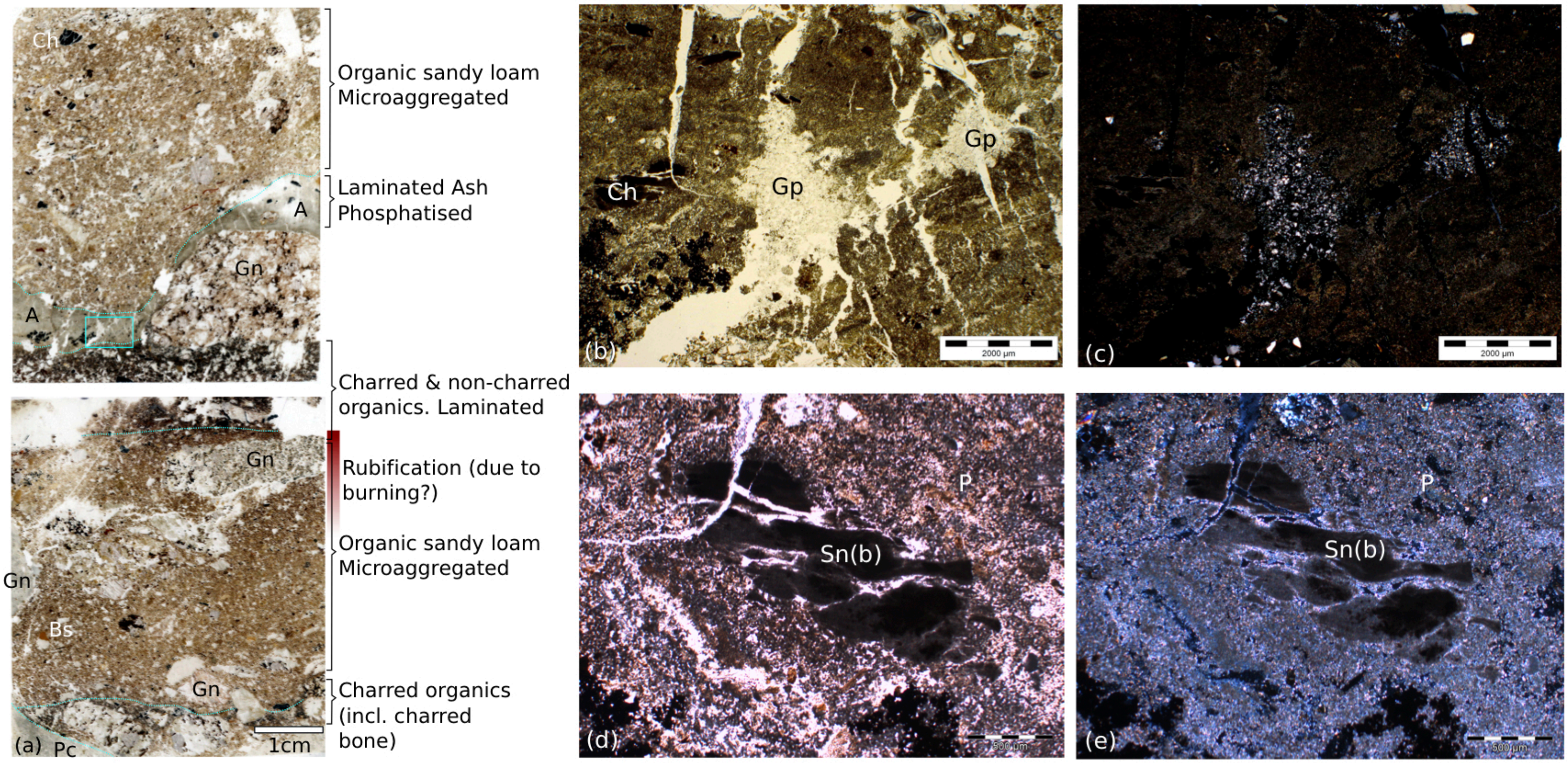

Supplementary Figure 4. Micromorphology of Phase D sediments. a: Contiguous sections across basal Phase D deposits (scanned thin sections) reveal microstratified human occupation layers with evidence for *in situ* burning. Frame shows location of (b) and (c). A: ash; Bs: burned sediment (intraclast); Ch: charcoal; Gn: gneiss clast; Gp: P: amorphous phosphates. b and c: Microlaminated phosphatised ash and charred organics (Ch). Phosphatisation of the ash and growth of gypsum (?) nodules (Gp) were probably linked with the diagenesis of bat guano. d and e: Charred and heat-fissured calcitic particle (Sn(b)), probably snail shell, in a matrix of ash-derived calcite and amorphous phosphate (P). Some of the calcitic matrix is recrystallized. b, d: PPL; c, e: XPL.

Supplementary Note 2. **Further detail relating to faunal analyses.**

A total of 14,485 bone fragments were analyzed from the approximately 150,000 specimens recovered during the 2010 and 2012 excavations of the cave. Of these, 16% were from the Late Pleistocene deposits, 45.7% from the Terminal Pleistocene (the most intense period of cave occupation), 31.8% from the Early Holocene, and 6.6% were from the Mid-Holocene layers. Supplementary Table 5 details the number of bone fragments studied per sedimentary context.

Bones are well preserved in all layers, with less than 1.4% of the specimens studied exhibiting moderate or heavy weathering (Supplementary Figure 6). Abraded bone fragments are also rare, with only 0.4% showing moderate to heavy abrasion. In Phase C, 2.1% exhibited evidence for hydrologic rounding, supporting the notion that the Late Pleistocene deposits consist mostly of redeposited habitation debris. In terms of bone fragment size, 83.4% of the specimens studied measured less than 30 mm, with an average bone fragment size of 22.35 mm (Supplementary Figure 7).

A total of 7622 specimens (52.6%) were identified to taxon (Supplementary Table 6). Small mammals dominate the assemblage, representing 95.5% of the total mammalian bone fragments studied and 95.2% of the mammalian specimens identified to taxon (Supplementary Table 7). In all phases of site occupation, small mammals account for more than 90% of the number of specimens. Large ungulates, including cervid, suids, and bovids are present all throughout the stratigraphy but in very small frequency (<4%). Fish, birds, and reptiles are present in slightly higher percentages. Non-mammalian fauna represent 13.5% and 5.9% of the specimens identified in Phase D and Phase C, respectively. Several reptile bone fragments, particularly varanids and *Python,* exhibit burning (58.2% NISP), suggesting that they were also utilized as a food source.

Supplementary Table 8 summarizes the vertebrate taxa recorded in Fa-Hien Lena. Cercopithecid monkeys dominate the faunal assemblage of the site, representing 54.9% of the total number of specimens identified to taxon. A total of 589 cercopithecid bone fragments from at least 27 individuals were recorded in the Late Pleistocene layers. These represent 48.7% of the specimens identified to taxon and 25.4% of the total number of faunal remains analyzed. Comparatively, a total of 2,138 cercopithecid bone fragments from at least 62 individuals (Supplementary Table 9) were identified from the Terminal Pleistocene deposits of the cave, representing 57.6% of the specimens identified to taxon. Deliberate targeting of cercopithecids by the people that occupied the cave continued until the Mid-Holocene, where cercopithecid fragments represent 61.2% of the number of identified specimens. In addition to deliberate targeting of taxa, there also appears to be deliberate targeting of a certain age class. Mortality profile based on dental wear[8, 9] suggests that prime–age adults were targeted from the Late Pleistocene to the Mid-Holocene (Supplementary Figures 8-9).

Three cercopithecid species are currently present in Sri Lanka: the cercopithecine *Macaca sinica* (toque macaque) and the colobine monkeys, *Trachypithecus vetulus* (purple-faced langur), and *Semnopithecus priam* (tufted gray langur). The three species occur sympatrically in Sri Lanka and all were identified in the faunal assemblage of Fa-Hien Lena. The cercopithecid specimens in the assemblage were differentiated based on morphology of certain dental[10-16] and skeletal elements[17-19]. Of the 4,188 cercopithecid bone fragments recorded in the site, 318 specimens (7.6%) were identified to species, with *M. sinica* (49.4%) being more common than *T. vetulus*

(34.6%) and *S. priam* (16%). In the Late Pleistocene layers, a total of 43 *M. sinica* specimens from at least five individuals were identified. This is in contrast to the number of *T. vetulus* (NISP= 23, MNI=3) and *S. priam* (NISP= 10, MNI=2) recorded from the same layers. But because the majority of the recorded cercopithecid specimens were long bone shaft fragments that cannot be assigned to species, all remains from a specific phase of site occupation were considered as comprising a single analytical assemblage.

In the Late Pleistocene layers, 10.9% of the identified remains are skull fragments (Supplementary Table 10), 1.9% are fragments of the mandible, 9.2% are isolated teeth (Supplementary Table 11), 5.3% are vertebrae (Supplementary Table 12), and 72.7% are appendicular skeletal elements (Supplementary Table 13). All skeletal elements, including carpal and tarsal bones, are present in the assemblage suggesting that complete carcasses were brought to the site (Supplementary Table 14). Vertebra fragments are slightly underrepresented due to preservation bias and the difficulty of assigning highly fragmented specimens to taxon. Supplementary Tables 15-29 details the number of cercopithecid dental and skeletal elements recorded in other phases of site occupation, and Supplementary Table 30-33 (see also Supplementary Figure 10) shows the MNE and MAU counts obtained for the cercopithecid skeletal elements in the assemblages.

A total of 64 skull fragments were recorded in the Late Pleistocene layers of the cave (Supplementary Table 10). These represent a minimum of seven crania, as suggested by the presence of seven left zygomatic bone fragments. Parietal and frontal bones are the most common skull elements in the assemblage but they are very fragmented and cannot be used to estimate the MNI/MNE. The mandible fragments (NISP= 11) from the Late Pleistocene layers of the site also account for at least seven individuals. The relatively equal number of skull and mandible fragments in the Late Pleistocene layers indicates that entire skulls were retained in the site. This is in contrast to the discrepancy observed between the number of skull and mandible fragments from Terminal Pleistocene layers of the site (Supplementary Table 25). A total of 264 skull fragments from at least 11 individuals (overlapping maxillae and frontal bones) were recorded in Phase C. By contrast, the 60 mandible fragments identified in the same layers represent at least 28 individuals. The same difference is observed when the number of isolated maxillary (NISP= 83, MNI= 7) and mandibular (NISP= 126, MNI= 14) teeth are considered. The paucity of monkey skulls in the Terminal Pleistocene layers of Fa-Hien Lena parallels the observations made by other authors[20-23] in their analyses of faunal assemblages from sites in Island Southeast Asia. Since no taphonomic process can account for the differential preservation of skulls and mandibles, they posit that monkey skulls could have been selectively retained, perhaps as trophies, by the people that settled the site.

The almost equal number of scapula (NISP= 15, MNI= 7) and pelvis (NISP= 17, MNI= 7) fragments in the Late Pleistocene layers of Fa-Hien Lena further supports the idea that entire monkey carcasses were being brought to the site. The same is true for the Terminal Pleistocene layers of the site which also yielded the same number of scapula and pelvis fragments (Supplementary Table 27).

Fragments of the appendicular skeleton, particularly of the long bones, were the most common specimens recorded in the site. In the Late Pleistocene layers, long bone fragments account for 36.7% of the cercopithecid specimens identified (Supplementary Table 14). The femur is the most common long bone represented in the Late Pleistocene assemblage, with a total of 82

identified fragments representing at least 28 elements, followed by the fibula (MNE= 13) and the tibia (MNE=12). There is an overrepresentation of hind limb over forelimb elements (78.9% difference in the MNE) in Phase D (Supplementary Table 30). This is not the case in the Terminal Pleistocene layers where there is only a 16.8% difference between arm and leg elements. All long bone elements are well represented in the Terminal Pleistocene and Early Holocene layers of the site. By contrast, hind limb and forelimb elements are overrepresented in the Late Pleistocene and mid-Holocene layers, respectively (Supplementary Figure 11). These differences are interpreted as being linked to changes in preference for skeletal element as material for bone tool production by the people that used the cave through time. Hind limb elements appear to have been selectively retained as materials for tool manufacture during the early phase of site occupation. Almost all of the osseous tools recovered from the Late Pleistocene layers of the site were manufactured from cercopithecid fibula and femur shaft fragments. By contrast, tools and artifacts made from both hind limb and forelimb elements were recorded in number in the Terminal Pleistocene and Early Holocene assemblages. During the mid-Holocene, there was a change in preference towards ulna shaft fragments as material for tool production, although tools from femur and fibula were also made.

Tool manufacture could also explain the high level of bone fragmentation observed in all phases of site occupation. Supplementary Table 34 details the degree of fragmentation of cercopithecid long bone elements as measured by NISP:MNE ratio[24] and fragment circumference/length completeness (see also Supplementary Figure 12). Only two complete cercopithecid skeletal elements were recorded in the assemblage studied, two fibulae from the Holocene layers. In the Late Pleistocene layers, 59.7% of the identified cercopithecid long bone fragments have less than half the original circumference and 73.1% measured less than half of the original length (i.e., Supplementary Figure 12). Fragments with preserved epiphyses are also very rare (NISP= 4). The femur is the most fragmented element, with 86.6% of the specimens having less than half of the original circumference. This is not surprising considering that manufacture of osseous tools from femur fragments involved breaking the bone into manageable segments before grinding them into the desired shape. This mode of tool production continued up to the Early Holocene, where 65.8% of the recorded femur fragments have less than half of the original circumference owing to the degree of working. Bone tools manufactured from femur shafts are rare in the mid-Holocene layers despite femur specimens being better preserved in these layers (76.5% of the specimens have the complete circumference).

Long bones of other small mammals are less fragmented compared to those of cercopithecids, particularly in the Late Pleistocene layers (Supplementary Figure 13). For instance, 64.3% of the sciurid long bone fragments recorded in Phase D have a complete circumference and 49.2% have preserved epiphyses. Mouse-deer and small carnivore long bones from the same layers are also well preserved. Again, these data support the idea that fragmentation of cercopithecid long bones relate to their use as the main material for bone tool manufacture. All osseous artifacts recorded in the Late Pleistocene layers of the cave were manufactured from cercopithecid long bone elements.

Butchery marks were recorded in nine specimens (0.39% of the total number of fragments) from the Late Pleistocene layers of the cave, mostly in small carnivore and squirrel bone fragments (Supplementary Table 35). Evidence for butchery was rare in cercopithecid bone fragments from Phase D, with only one cutmarked specimen recorded (a femur shaft fragment). By contrast, all

the viverrid and otter remains recorded in Phase D had cutmarks. For example, an otter (*Lutra lutra*) proximal humerus shaft with cutmarks on the posterolateral surface (Supplementary Figure 4B) was recovered from context 253, the earliest deposit in the site. Two squirrel long bone fragments with cutmarks were also recorded from Phase D (i.e. tibia shaft in Supplementary Figure 4A). The frequency of bones with butchery marks is slightly higher in the Terminal Pleistocene layers (0.98% of the total number of fragments) of the site. Similar to Phase D, however, cercopithecid bone elements with evidence for butchery were also rare in the Terminal Pleistocene layers. From the 55 bone fragments with butchery marks, only three were from cercopithecids. This is despite the presence of complete epiphyses where cutmarks are usually observed[22, 23, 25, 26]. Like in Phase D, most of the cutmarks were recorded in giant squirrel (N=20) and viverrid/mustelid (N=23) femur and humerus specimens.

The locations of the cutmarks on the sciurid and small carnivore long bones were also consistent. In the femur, all the cutmarks were observed on the lateral and medial shaft near the distal articular end. On the humerus, the cutmarks were concentrated on the lateral/medial supracondylar ridge and on the area just above the lateral/medial epicondyle. The prevalence of cutmarked distal humerus fragments (40% of specimens with cutmarks) suggests a systematic butchery process that involved the disarticulation of the ulna from the humerus. Little can be said about the cercopithecid carcass processing sequence during the Late/Terminal Pleistocene phases of the site because of the low frequency of skeletal elements with traces of butchery. Severe fragmentation of skeletal elements in the Late Pleistocene layers, in addition to high frequency of burning (see below), obscures the evidence for butchery. But this is not the case in the Terminal Pleistocene layers where there is better skeletal element preservation.

As mentioned earlier, fragments of bone finished tools, blanks, and waste pieces, were recorded in all phases of occupation suggesting the use of the cave as a site for osseous tool manufacture. The bone tools recovered from the Late Pleistocene layers of Fa-Hien Lena were all manufactured from cercopithecid long bones, except for a worked macaque canine recorded from context 253. A total of 36 specimens with surface modifications consistent with bone tool manufacture were recorded in the Late Pleistocene layers of the site (1.3% of the total number of specimens studied). These include ten fragments of finished tools (i.e., those retaining diagnostic morphology, including tips of bone points/spatulas). The rest are fragments which represent either waste pieces or tool blanks.

Burning and calcination were the most common modifications observed in bone fragments from the Fa-Hien Lena assemblage (Supplementary Table 35). Burning was recorded in skeletal elements of all the taxa identified, including fish and reptiles. Indeed, burning and calcination were the only modifications recorded in skeletal elements of non-mammalian taxa. The amount of burnt and calcined fish remains is high with 77.8% of the identified remains burnt/calcined. By comparison, 58.2% of snake and lizard remains exhibited burning. A significant proportion of sciurid remains also exhibited evidence for burning (31%). This is in comparison to the 16.1% recorded burnt cercopithecid remains. The patterns of burning, including the presence of burning marks in the inner skull as well as completely burnt /calcined carpals and tarsals, suggest that burning occurred after skeletal disarticulation and deposition.

Burnt and calcined specimens were recorded in all phases of site occupation, although they appear to be more common in the Late Pleistocene layers in terms of relative proportion. A total of 273 burnt bone and tooth fragments were recorded in Phase D, representing 11.8% of

specimens recovered. In addition, 12.1% of the specimens from these layers were either partially (N=179) or completely (N=102) calcined. In context 253, the oldest layer in the site, 27.7% of the recorded bone fragments were burnt or calcined. By comparison, 12.8% of the specimens from the Terminal Pleistocene layers showed evidence for burning and 7.1% were calcined. The proportion of burnt fragments was slightly lower in the Early (9.6%) and Mid-Holocene layers (5.1%) of the site.

Next to cercopithecids, sciurids were the most common taxa identified in the faunal assemblage of Fa-Hien Lena. A total of 2026 sciurid remains were recorded in the site, representing 26.6% of the total number of identified specimens and 13.9% of the fragments analyzed. Sciurid remains represent 27.6% of the specimens identified from the Late Pleistocene Layers, 27.1% of the specimens from the Terminal Pleistocene layers, and 28.8% and 10.2% of those from the Early and Mid-Holocene assemblages respectively. At least three squirrel taxa were identified in the assemblage based on the morphology of teeth and certain post-cranial elements: the giant grizzled squirrel *Ratufa macroura* and the flying squirrels *Petinomys* and *Petaurista*. The palm squirrel *Funambulus* might also be present in the assemblage, as some diagnostic elements including maxillae and mandibles were identified in layers not included in the present study (i.e., non-dated sedimentary contexts). Specimens that cannot be securely assigned to genus were identified to the family level.

Although both taxa were recorded in all the phases of site occupation, it appears that the people that utilized the site targeted *Ratufa* as a prey more than the flying squirrels. For instance, a total of 121 (36.2%) grizzled giant squirrel remains from at least 16 individuals were identified from the Late Pleistocene layers compared to the 20 *Petinomys/Petaurista* skeletal and dental elements from at least three individuals recorded from the same layers. Similarly, 41.6% of the sciurid remains from the Terminal Pleistocene layers were assigned to *R. macroura* and 4.3% were identified as from flying squirrels. *Petaurista* and *Petinomys* are both nocturnal, arboreal species. On the other hand, the grizzled giant squirrel, like the cercopithecid taxa identified in the assemblage, is a diurnal, arboreal species. Because *Ratufa* and the cercopithecids occur in the same forest environment, it is most likely that they were encountered together by the people that settled the site. All sciurid skeletal elements were represented in all phases of site occupation (Supplementary Tables 36-39, Supplementary Figure 14) indicating that entire carcasses were brought to the site. A high proportion of sciurid remains from the Late and Terminal Pleistocene levels were burnt and calcined (30.2% and 27.9% respectively). For instance, Supplementary Figure 15 shows *R. macroura* calcanei in different stages of burning and calcination. The presence of cutmarks on long bone epiphyses indicates disarticulation and division of carcasses into smaller units during butchery. Implements made from squirrel skeletal elements were rare (N=4, modified femur) and were only recorded in the Early and Mid-Holocene levels.

Other small mammals recorded in the Fa-Hien Lena assemblage include six different small carnivore taxa from four different families. Vivverids, the most common small carnivore in the assemblage, were represented by two genera: the palm civet *Paradoxurus* and the Indian civet *Viverricula*. Theses taxa were recorded in all phases of site occupation but in much lower frequencies compared to sciurids and cercopithecids. Herpestids and mustelids were also recorded from the Late Pleistocene to the Early Holocene layers of the site. As pointed out before, numerous small mammal long bone fragments exhibited evidence for butchery, as well as burning and calcination, suggesting that they were probably consumed. However, their low

frequency suggests that they did not play a major role in the subsistence of the people that utilized the site.

In addition to small carnivores, mouse deer (*Moschiola)* remains were also observed in all occupational phases of Fa-Hien Lena. The mouse deer skeletal elements did not yield any evidence for butchery, unlike those from the small carnivores. However, a significant proportion was burnt or calcined. For instance, 10 out of the 14 mouse deer specimens recorded from the Late Pleistocene layers were burnt. In the Terminal Pleistocene layers, 72% of the mouse deer remains exhibited evidence for burning or calcination. Like the small carnivores, mouse deer were probably utilized by the people that settled Fa-Hien Lena as a supplementary source of protein.

Large ungulates, including cervids, bovids, and suids, were recorded in all phases of site occupation, but in very low frequency. Elephants and rhinoceros were also identified, albeit only in the Terminal Pleistocene and Early Holocene layers. Interestingly, large ungulates were mostly represented in the assemblage by tooth or long bone fragments, suggesting that they were not butchered in the site. For instance, tooth fragments represent 21 out of the 31 suid specimens identified in the Terminal Pleistocene layers. Likewise, 56.6% of the cervid specimens from the same layers were molar/premolar fragments. At least two deer taxa were noted based on dental size. A complete lower third molar from the Terminal Pleistocene layers allowed the identification of the smaller taxon as the barking deer *Muntiacus muntjak*. The larger taxon cannot be assigned to genus because of the fragmentary nature of the specimens. Two large deer species are currently present in Sri Lanka: the spotted deer (*Axis* sp.) and the sambar deer (*Rusa unicolor*).

Elephants were also represented by tooth fragments, including a completely burnt neonate molar from the Terminal Pleistocene layers. The presence of a rhinoceros upper molar fragment in Phase B indicates the presence of this species as far south as Sri Lanka's Western Province during the early Holocene. In the Late Pleistocene layers, large ungulates were also represented mostly by tooth fragments. For instance, two burnt bovid molar fragments were identified from a sediment layer dated to 39,876-38,490 cal. BP. Long bone fragments, the majority of which were worked, constitute the rest of the ungulate remains recorded in the site. Worked deer metapodials and antler fragments (N= 16) were recorded in the Terminal Pleistocene and Early Holocene layers of the site. The low frequency of large ungulate remains in the faunal assemblage suggests that they did not play a significant role in the subsistence of the people that settled the site. Instead, large ungulate skeletal and dental elements were most likely imported to the site as finished tools or as materials for tool production as evidenced by the high proportion of worked fragments.

Reptiles and fish were also recorded in all phases of site occupation. The fish remains from Fa-Hien Lena are represented by two families, silurids (catfish) and cyprinids (carps), identified from cranial elements. Complete pharyngeal teeth allowed the identification of the cyprinid in the assemblage as representing the genus *Tor* (mahseer). Nonetheless, 71.5% of the fish remains recorded in the site are spine and vertebra fragments that cannot be identified to taxon. Butchery marks are usually rare in fish bones from archaeological sites[27, 28], and the fish remains from Fa-Hien Lena did not exhibit any evidence for butchery/processing. Anthropic modifications in fish bones from the site were restricted to burning. 77.8% of the total number of fish remains identified in the site were burnt or calcined. Interestingly, all fish bones from the Late

Pleistocene layers of the site exhibited evidence for burning (e.g., Supplementary Figure 16). Reptiles account for 4.4% of the total number of identified specimens from the Late Pleistocene layers and 5.9% of those from the Terminal Pleistocene. Reptiles are represented by pythons, colubrid snakes, water monitors, and dragon lizards as well as geckos and skinks. Crocodiles were recorded in the assemblage; but only in the Terminal Pleistocene layers. Butchery marks were not observed in any of the reptile skeletal elements examined and similar to fish bones, anthropic modifications were restricted to burning. 28.8% of the reptile remains identified in the site were burnt or calcined. In the Late Pleistocene layers, 74.1% of the monitor lizard remains and 85.7% of the snake specimens exhibited burning.

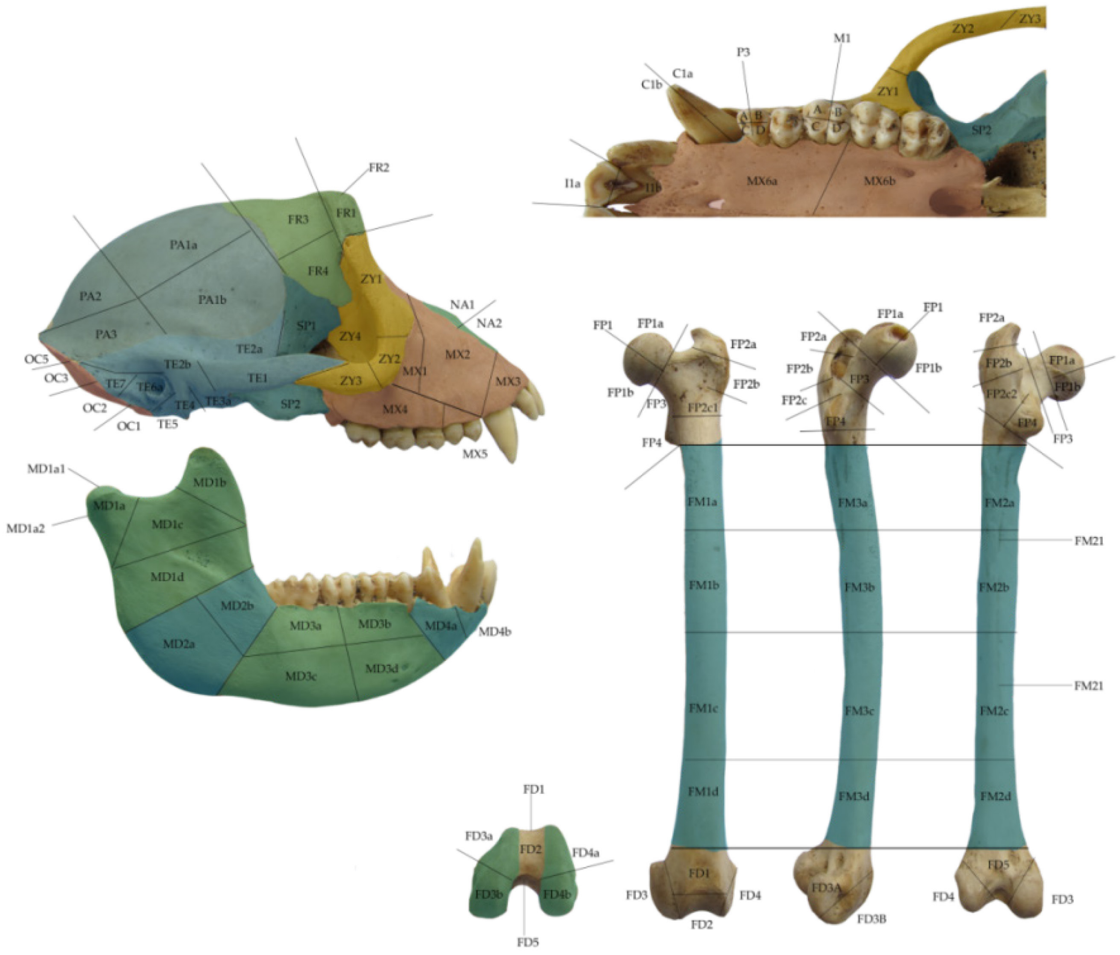

Supplementary Figure 5. Example of the system for recording bone fragments used in the current study.

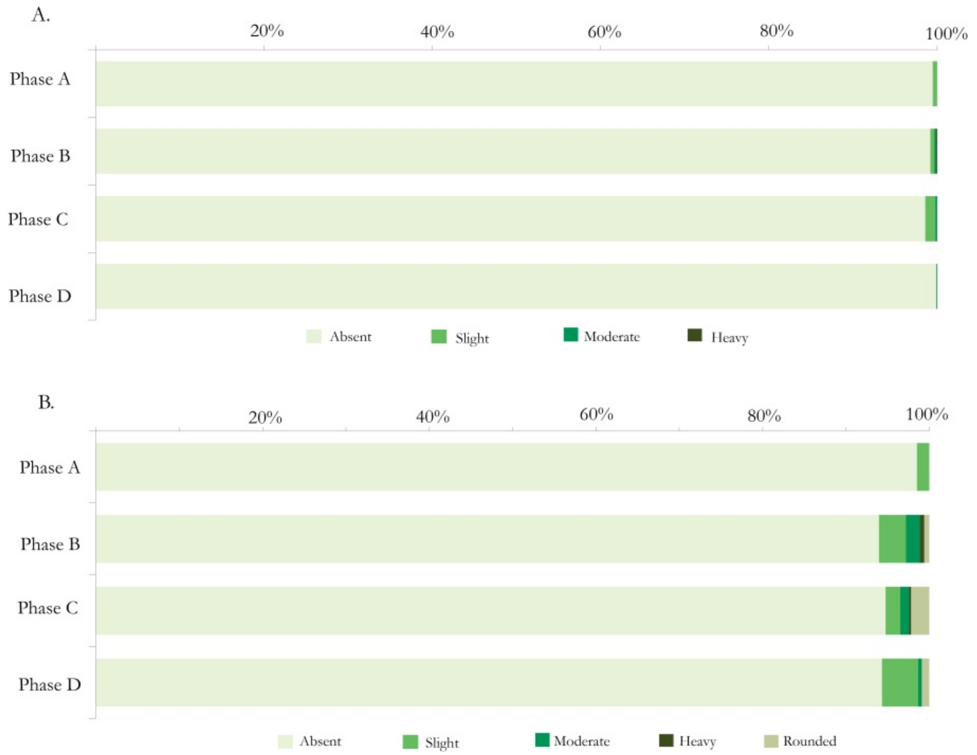

Supplementary Figure 6. Distribution of bone fragments based on abrasion (**A**) and weathering (**B**) in the different occupational phases Fa-Hien Lena.

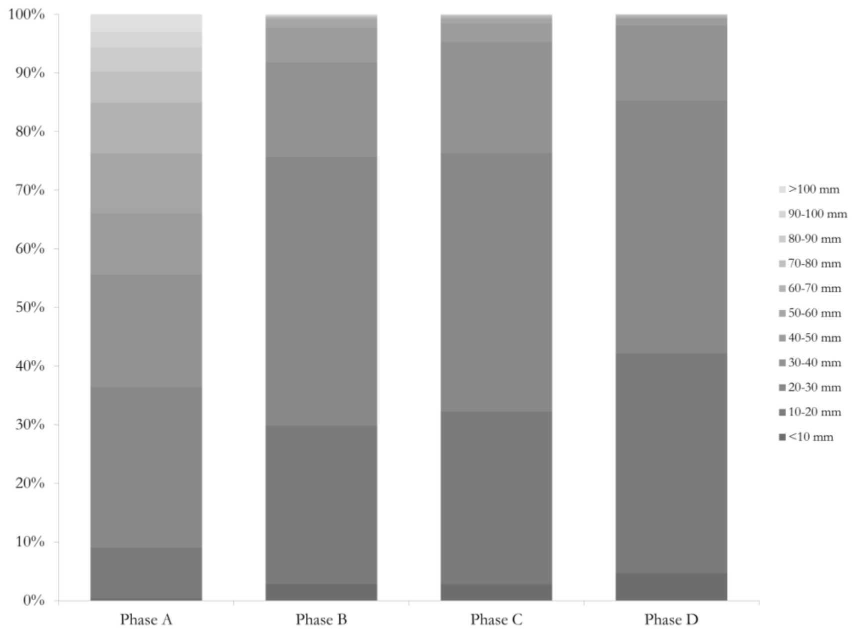

Supplementary Figure 7. Fragmentation (based on fragment length) of specimens recorded in the different occupational phases of Fa-Hien Lena.

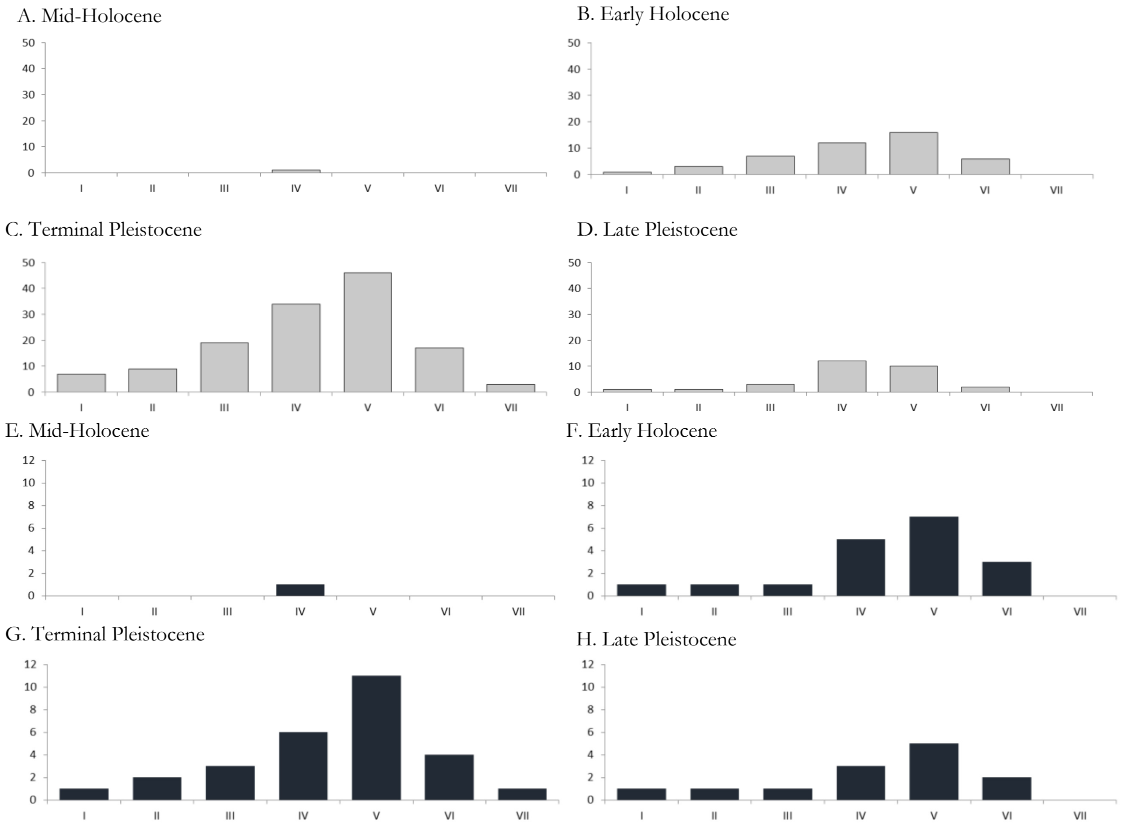

Supplementary Figure 8. Mortality profiles of cercopithecid monkeys (following ref. [8]) considering the NISP (**A-D**) and the MNI (**E-H**).

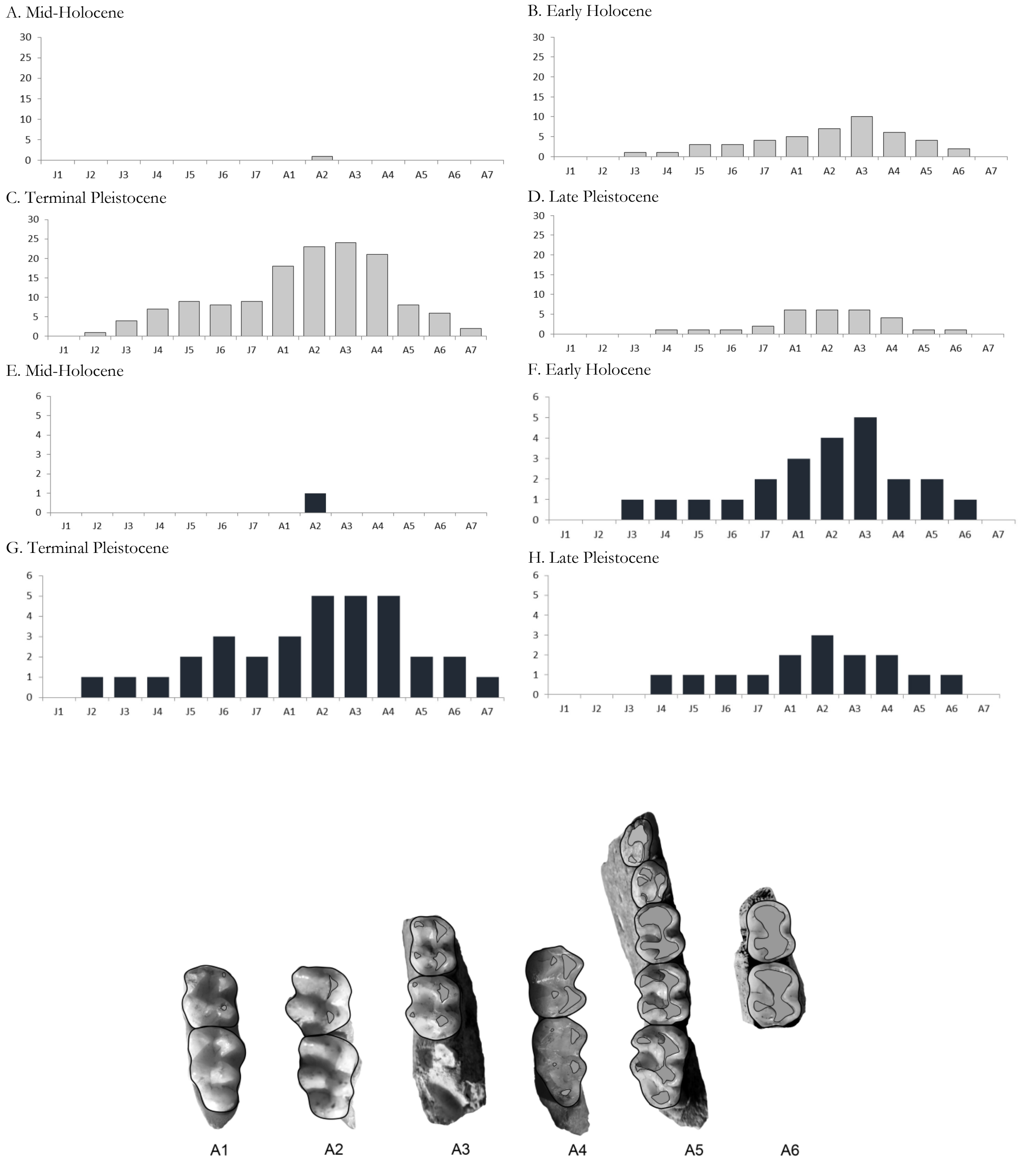

Supplementary Figure 9. Mortality profiles of cercopithecid monkeys (following ref. [9]) considering the NISP (**A-D**) and the MNI (**E-H**).

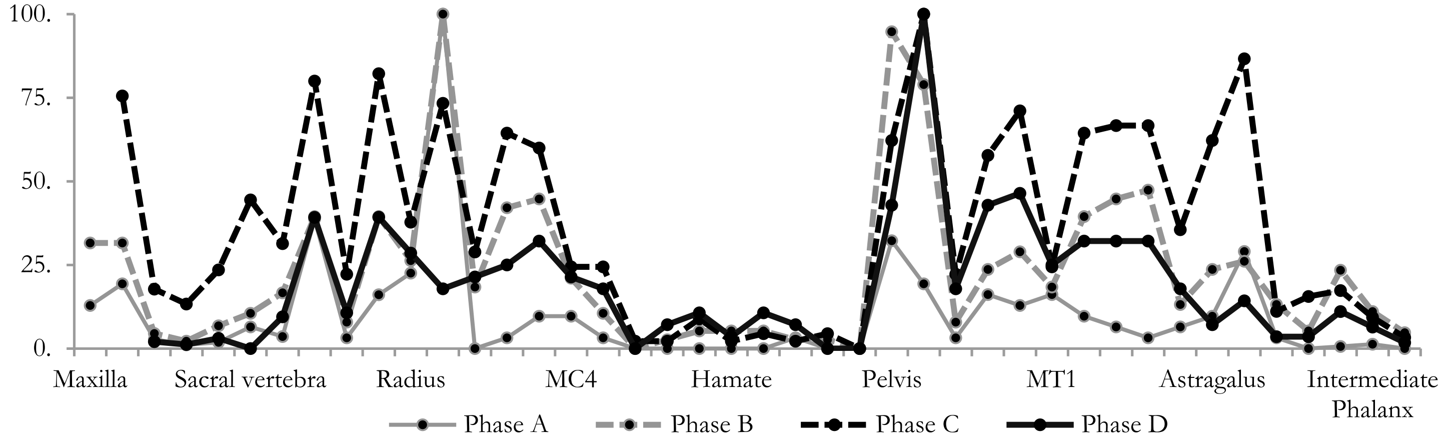

Supplementary Figure 10. Body part representation (% MAU) of cercopithecids in the different phases of occupation of Fa-Hien Lena.

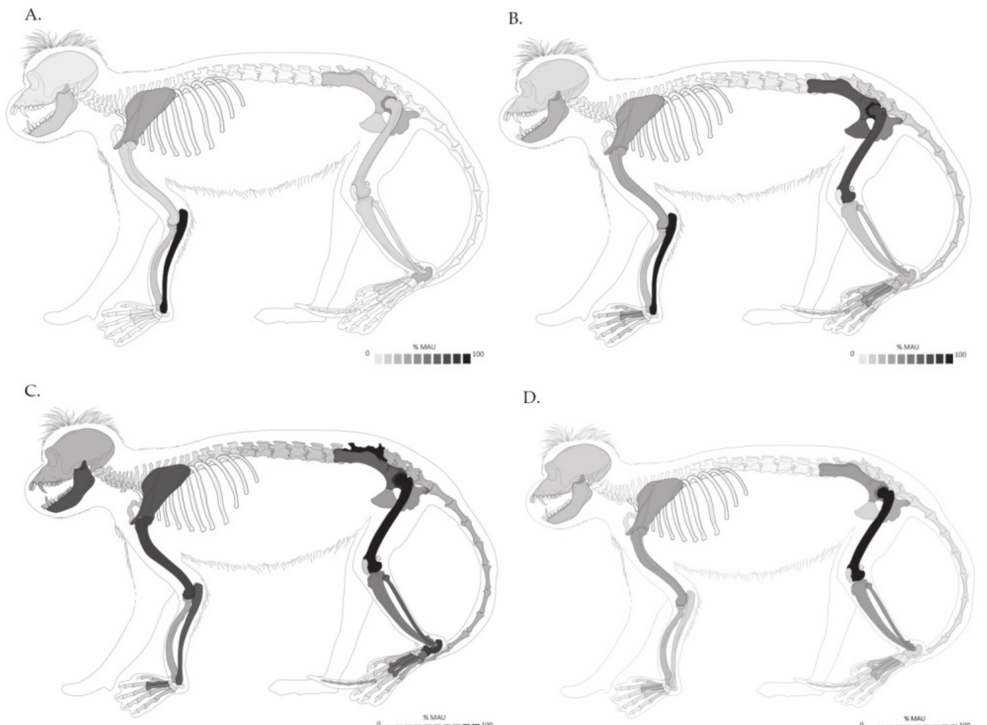

Supplementary Figure 11. Cercopithecid skeletal element representation in Fa-Hien Lena considering the % Minimum Number of Animal Unit (%MAU). **A**- Phase A, **B**- Phase B , **C**- Phase C, **D**- Phase D.

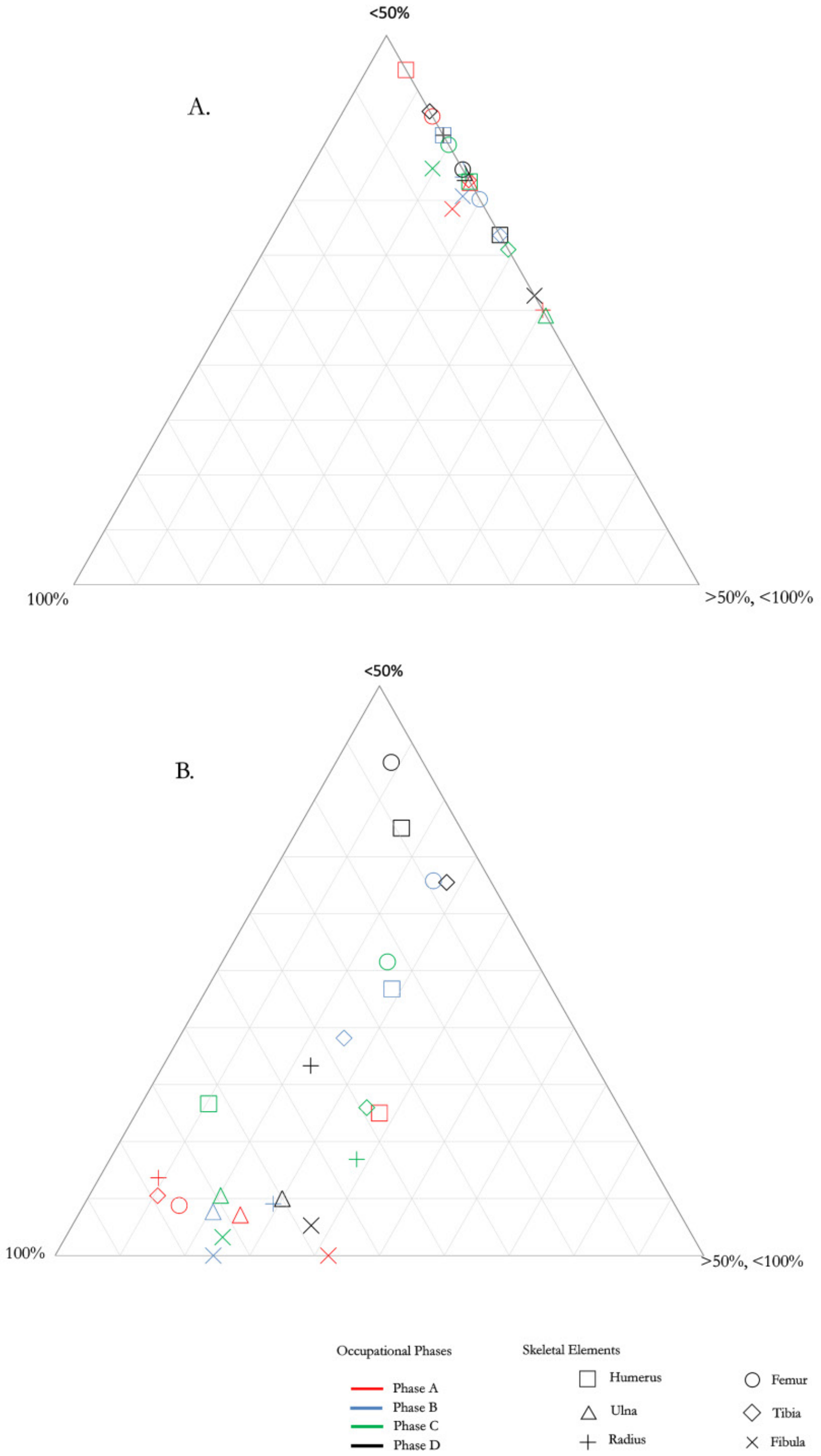

Supplementary Figure 12. Ternary scatter plot illustrating cercopithecid long-bone length (**A**) and circumferential completeness (**B**).

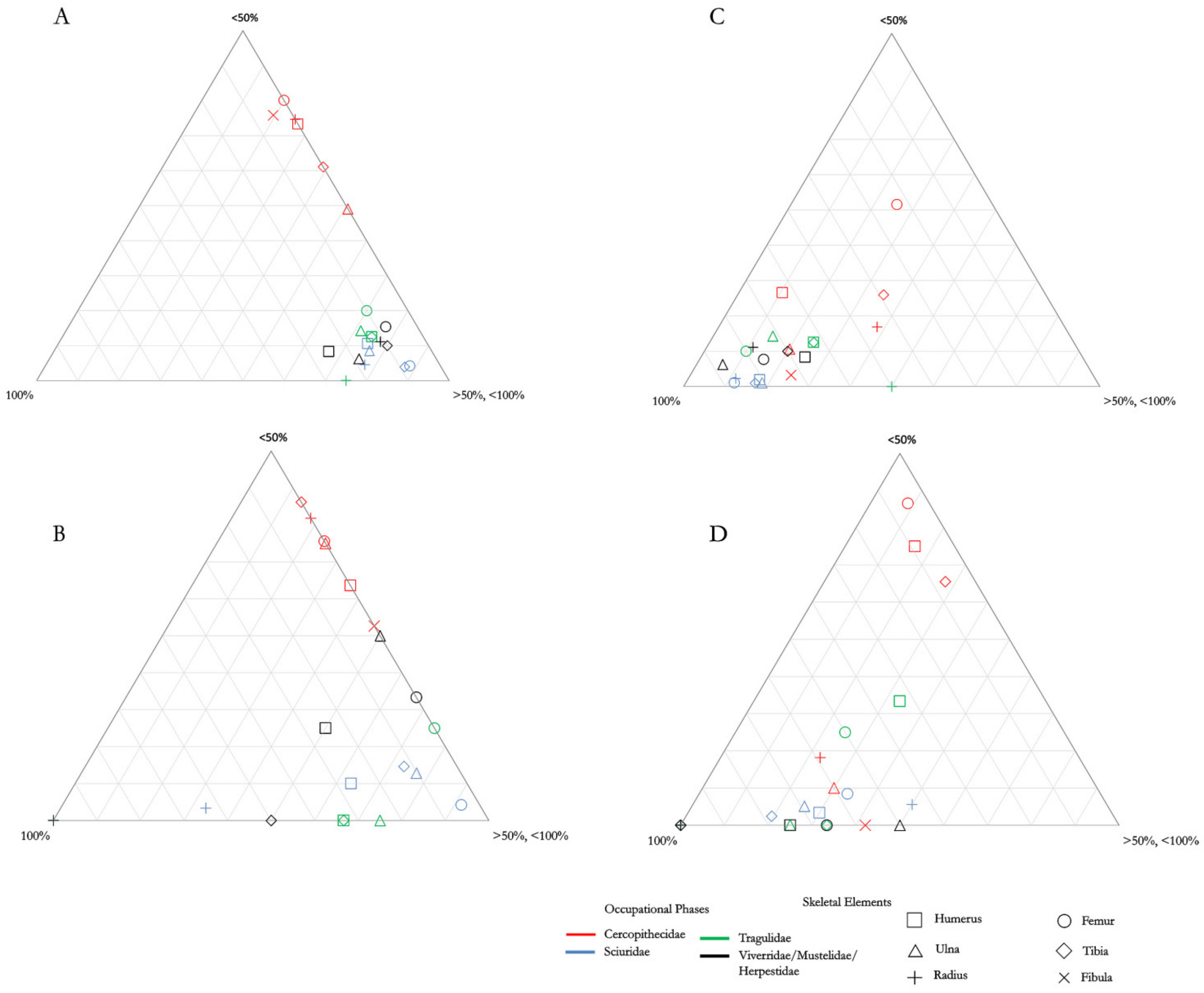

Supplementary Figure 13. Ternary scatter plot illustrating small mammal long-bone fragmentation (**A**- Terminal Pleistocene, length completeness; **B**- Late Pleistocene, length completeness; **C**- Terminal Pleistocene, circumferential completeness; **D**- Late Pleistocene, circumferential completeness).

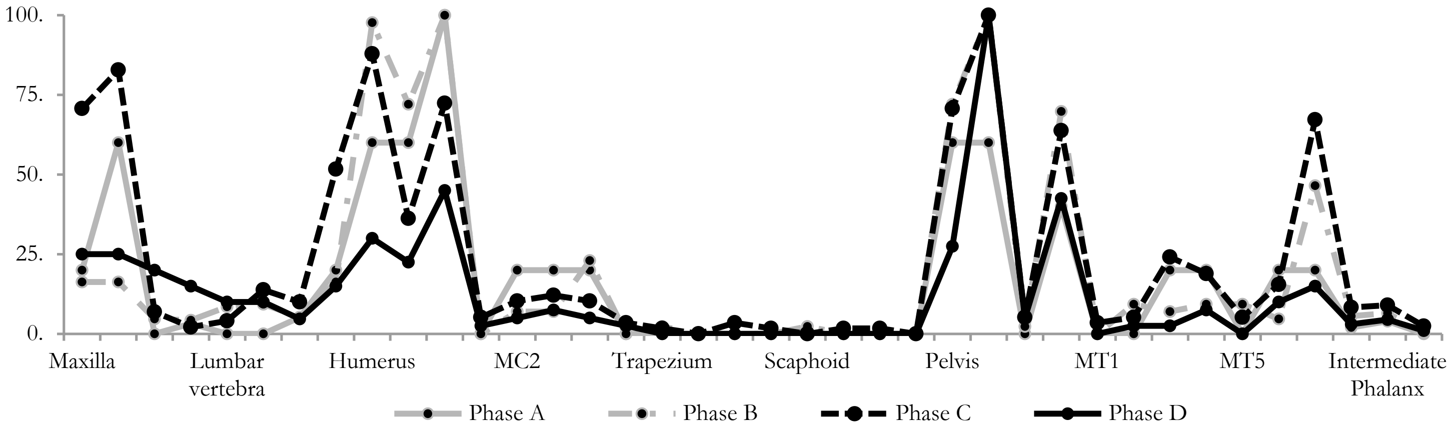

Supplementary Figure 14. Body part representation (% MAU) of sciurids in the different phases of occupation of Fa-Hien Lena.

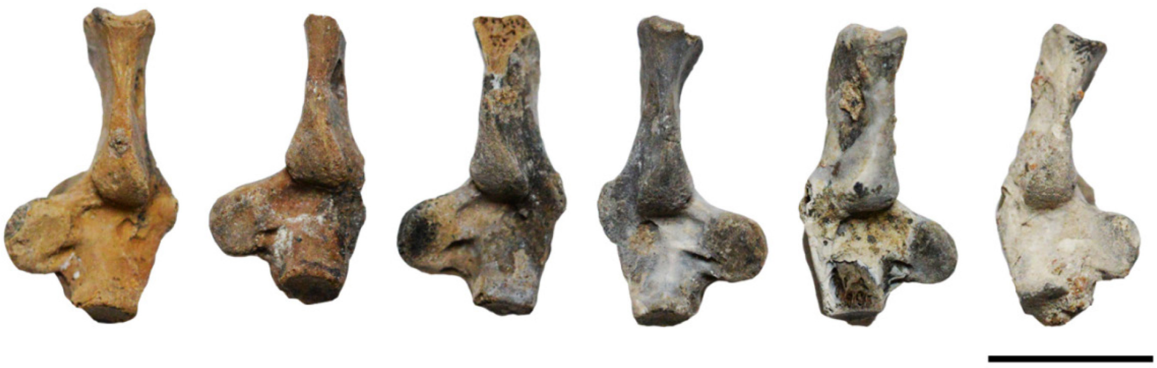

Supplementary Figure 15. *Ratufa macroura* calcanei in different stages of burning/calcination from the Late Pleistocene deposits of Fa-Hien Lena (measurement bar= 1 cm).

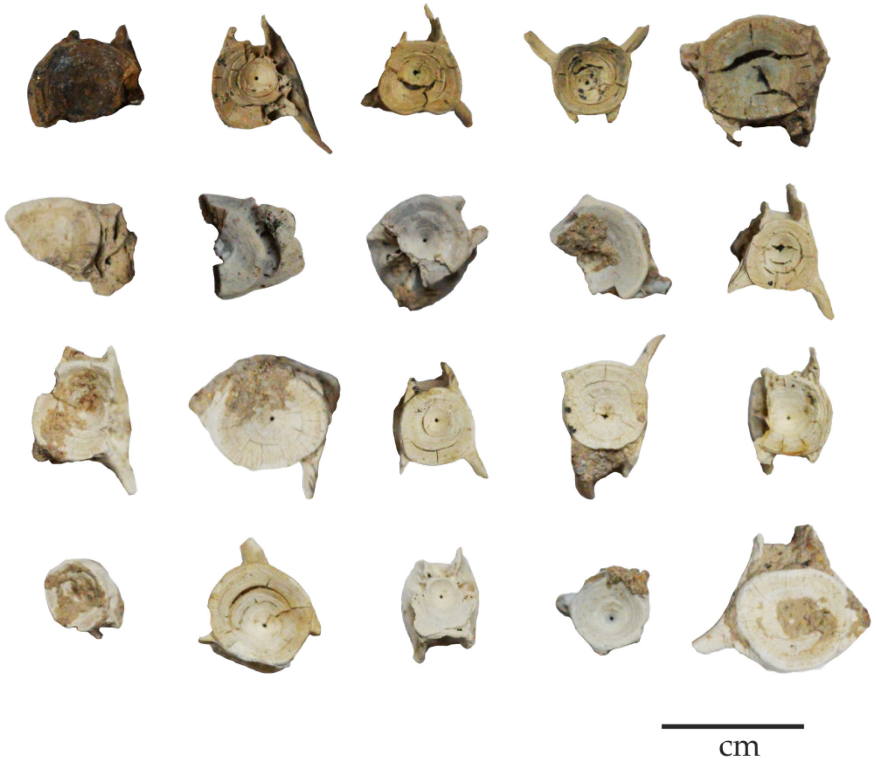

Supplementary Figure 16. Fish vertebrae in different stages of burning/calcination from the Late Pleistocene deposits of Fa-Hien Lena.

Supplementary Table 5. Number of bone fragments from different sedimentary contexts that were included in the current study

Phase	Context	Date	Number of fragments analyzed	Total number of fragments
A	216		661	956
	131		114	
	31/32/135	5653-5488 cal. BP.	181	
B	7/151	7955-7791 cal. BP.	62	4595
	52/153		451	
	170		188	
	116/128		2281	
	180		380	
	136	7935-7762 cal. BP.	535	
	38/206		193	
	138	8595-8430 cal. BP.	210	
	98		35	
	107		260	
C	168		87	6615
	237		93	
	139/140	12,419-12,062 cal. BP.	576	
	141	12,530-12,120 cal. BP.	94	
	173		605	
	142	12,590-12,236 cal. BP.	207	
	248		2354	
	174/247	12,575-12,150 cal. BP.	2294	
	144/161/164	12,386-11,910 cal. BP.	245	
	163/235	13,085 +/- 75	60	
D	108		318	2319
	110		414	
	118		238	
	145	37,912-36,300 cal. BP.	36	
	179		132	
	175	39,876-38,490 cal. BP.	288	
	157		55	
	158	40, 250 +/- 650	64	
	159	48,046-45,028 cal. BP.	31	
	165	42,800 +/- 900	38	
	253		705	
Total				14,485

Supplementary Table 6. Number of identified specimens (NISP) in the different occupational phases of Fa-Hien Lena.

	Phase A	Phase B	Phase C	Phase D
Unidentified Bone Fragments	467	2384	2903	1109
NISP	489	2211	3712	1210
Percentage NISP	51.2	48.11	56.1	52.2

Supplementary Table 7. Distribution of mammalian taxa grouped by body size in the different occupational phases of Fa-Hien Lena.

Unidentified Mammalian Bone Fragments	Phase A	Phase B	Phase C	Phase D
Micromammals- 100g to 1 kg	11	18	4	16
Small mammals- 1 kg to 25 kg	447	2325	2772	1059
Large mammals class 1- 25 kg to 200 kg	8	40	112	22
Large mammals class 2- 200 kg to 1000 kg	1	1	12	10
Large mammals class 3: >1000 kg	0	0	3	2
Total Unidentified Bone Fragments	467	2384	2903	1109
Identified Mammalian Bone Fragments	Phase A	Phase B	Phase C	Phase D
Micromammals- 100g to 1 kg	8 (2.12%)	65 (3.28%)	74 (2.12%)	99 (9.46%)
Small mammals- 1 kg to 25 kg	360 (95.24%)	1884 (95.10%)	3275 (93.84%)	936 (89.40%)
Large mammals class 1- 25 kg to 200 kg	8 (2.12%)	29 (1.46%)	125 (3.58%)	10 (0.96%)
Large mammals class 2- 200 kg to 1000 kg	2 (0.53%)	1 (0.05%)	15 (0.43%)	2 (0.19%)
Large mammals class 3: >1000 kg	0	2 (0.10%)	1 (0.03%)	0
Total Mammals (NISP)	378	1981	3490	1047
Total Mammalian Bone Fragments	Phase A	Phase B	Phase C	Phase D
Micromammals- 100g to 1 kg	19 (2.25%)	83 (1.9%)	78 (1.22%)	115 (5.33%)
Small mammals- 1 kg to 25 kg	807 (95.5%)	4209 (96.43%)	6047 (94.59%)	1995 (92.53%)
Large mammals class 1- 25 kg to 200 kg	16 (1.89%)	69 (1.58%)	237 (3.71%)	32 (1.48%)
Large mammals class 2- 200 kg to 1000 kg	3 (0.36%)	2 (0.05%)	27 (0.42%)	12 (0.56%)
Large mammals class 3: >1000 kg	0	2 (0.05%)	4 (0.06%)	2 (0.09%)
Total	845	4365	6393	2156

Supplementary Table 8. Number of identified specimens of the different vertebrate taxa identified in Fa-Hien Lena.

Class	Order	Family	Taxon	Common Name	Phase A		Phase B		Phase C		Phase D	
					NISP	% NISP	NISP	% NISP	NISP	% NISP	NISP	% NISP
Actinopterygii					4	0.82	39	1.76	17	0.46	43	3.55
	Cypriniformes	Cyprinidae	*Tor* sp.	Mahseer	-	-	13	0.59	10	0.27	5	0.41
	Siluriformes	Siluridae		Catfish	-	-	5	0.23	6	0.16	2	0.17
Amphibia	Anura				3	0.61	-	-	3	0.08	42	3.47
Aves					1	0.2	5	0.23	7	0.19	-	-
	Apodiformes	Apodidae		Swift	1	0.2	1	0.05	2	0.05	-	-
	Passeriformes				-	-	-	-	-	-	-	-
		Hirundinidae		Swallow	-	-	1	0.05	-	-	-	-
		Corvidae		Crow/Magpie	-	-	2	0.09	2	0.05	-	-
		Sturnidae		Starling	-	-	1	0.05	-	-	-	-
	Galliformes	Phasianidae	*Gallus* sp.	Junglefowl	2	0.41	4	0.18	12	0.32	-	-
	Strigiformes	Strigidae		Owl	1	0.2	1	0.05	1	0.03	-	-
Reptilia	Crocodilia	Crocodylidae	*Crocodylus* sp.	Crocodile	-	-	-	-	4	0.11	-	-
	Testudines	Bataguridae		Terrapin	-	-	-	-	-	-	1	0.08
	Squamata	Varanidae	*Varanus* sp.	Monitor lizard	18	3.68	47	2.13	73	1.97	27	2.23
		Agamidae		Agamid lizards	6	1.23	10	0.45	6	0.16	6	0.5
		Geckonidae/Scinicidae		Gecko/Skink	6	1.23	1	0.05	-		2	0.17
		Pythonidae	*Python* cf. *molurus*	Indian Python	21	4.29	41	1.85	43	1.16	11	0.91
		Colubridae/Viperidae		Colubrid/Viper	48	9.82	59	2.6	36	0.97	24	1.98

Supplementary Table 8. continued

Class	Order	Family	Taxon	Common Name	Phase A		Phase B		Phase C		Phase D	
					NISP	% NISP	NISP	% NISP	NISP	% NISP	NISP	% NISP
Mammalia	Primates	Cercopithecidae			265	54.2	1071	48.43	2021	54.45	513	44.87
			Macaca sinica	Toque macaque	18	3.68	41	1.86	55	1.48	43	1.9
			Trachypithecus vetulus	Purple-faced langur	11	2.25	35	1.58	41	1.11	23	1.07
			Semnopithecus priam	Tufted gray langur	5	1.02	15	0.68	21	0.57	10	0.83
		Lorisidae	*Loris*	Loris	-	-	-	-	3	0.08	-	-
	Rodentia	Hystricidae	*Hystrix* cf. *indica*	Indian porcupine	2	0.41	8	0.36	23	0.62	6	0.5
		Sciuridae			21	4.29	332	15.02	545	14.68	193	15.94
			Ratufa. macroura	Grizzled giant squirrel	24	4.91	269	12.17	418	11.26	121	10
			Petinomys/*Petaurista*	Flying squirrel	5	1.02	35	1.58	43	1.16	20	1.64
		Muridae			1	0.2	16	0.72	17	0.46	59	4.88
	Lagomorpha	Leporidae	*Lepus* cf. *nigricollis*	Indian hare	-	-	4	0.18	10	0.27	-	-
	Soricomorpha	Soricidae		Shrew	-	-	1	0.05	2	0.05	1	0.08
	Chiroptera				2	0.41	10	0.45	5	0.13	12	0.99
		Hipposideridae	*Hipposideros* sp.	Leaf-nosed bat	-	-	-	-	2	0.05	2	0.17
		Megadermatidae	*Megaderma* sp.	False vampire bat	-	-	1	0.05	3	0.08	1	0.08
		Rhinolophidae	*Rhinolophus* sp.	Horshoe bat	-	-	2	0.09	2	0.05	3	0.25
		Pteropodidae	*Cynopterus* sp.	Short-nosed fruit bat	-	-	-	-	-	-	1	0.08
			Pteropus sp.	Flying fox	-	-	11	0.49	3	0.08	2	0.17

Supplementary Table 8. continued

Class	Order	Family	Taxon	Common Name	Phase A		Phase B		Phase C		Phase D	
					NISP	% NISP	NISP	% NISP	NISP	% NISP	NISP	% NISP
Mammalia	Carnivora	Viverridae			4	0.82	32	1.45	46	1.24	5	0.41
			Paradoxurus sp.	Palm civet	3	0.61	5	0.23	18	0.48	3	0.25
			Viverricula sp.	Indian civet	1	0.2	2	0.09	2	0.05	-	-
		Herpestidae	*Herpestes*	Mongoose	-	-	8	0.36	6	0.16	2	0.17
		Mustelidae	*Lutra lutra*	Otter	-	-	3	0.14	17	0.46	1	0.08
		Felidae	*Prionailurus* sp.	Spotted/Fishing cat	-	-	-	-	1	0.03	-	-
			Felis sp.	Jungle cat	-	-	-	-	2	0.05	-	-
		Canidae	*Canis* cf. *aureus*	Golden jackal	-	-	4	0.18	11	0.3	-	-
	Artiodactyla	Tragulidae	*Moschiola* sp.	Chevrotain	6	1.23	48	2.17	43	1.16	14	1.16
		Suidae	*Sus scrofa*	Boar	1	0.2	4	0.18	31	0.84	2	0.17
		Cervidae			3	0.61	6	0.27	36	0.97	3	0.25
			Rusa unicolor /*Axis* sp.	Deer	2	0.41	7	0.32	19	0.51	3	0.25
			Muntiacus muntjak	Barking deer	2	0.41	8	0.36	28	0.75	2	0.17
		Bovidae			2	0.41	1	0.05	15	0.4	2	0.17
	Perissodactyla	Rhinocerotidae	*Rhinoceros* sp.	Rhinoceros	-	-	1	0.05	-		-	-
	Proboscidea	Elephantidae	*Elephas maximus*	Asian elephant	-	-	1	0.05	1	0.03	-	-

Supplementary Table 9. Number of Identified Specimens (NISP) Minimum Number of Individuals (MNI) of cercopithecids and sciurids in the different occupational phases of Fa-Hien Lena.

Period	Context	Cercopithecids		Sciurids	
		NISP	MNI	NISP	MNI
A	216	230	14	16	2
	131	22	2	6	1
	31/32/135	47	3	28	2
B	7/151	11	2	22	2
	52/153	107	5	69	3
	170	34	2	32	2
	116/128	460	12	242	11
	180	75	3	66	3
	136	124	5	74	4
	38/206	5	1	31	2
	138	123	3	62	4
	98	2	1	5	1
	107	221	6	43	2
C	168	25	3	15	2
	237	48	3	14	3
	139/140	181	7	56	3
	141	47	2	21	2
	173	103	6	189	9
	142	12	1	21	2
	248	847	14	308	13
	174/247	696	16	331	14
	144/161/164	146	8	45	3
	163/235	33	2	6	2
D	108	104	4	65	3
	110	160	5	67	3
	118	46	3	28	2
	145	2	1	25	2
	179	28	2	20	2
	175	77	3	32	2
	157	2	1	9	1
	158	6	1	4	1
	159	3	1	1	1
	165	8	1	1	1
	253	153	5	82	5

The MNI values obtained when considering specific sedimentary contexts are much higher compared to the estimates derived considering entire assemblages[24].

Supplementary Table 10. Cercopithecid skull and mandible fragments recovered from the Late Pleistocene layers (Phase D) of Fa-Hien Lena.

	NISP				Total
	Right	Left	R/L	Indet.	
Skull Fragments					64
Maxilla	7	2	/	/	9
Frontal	7	6	/	4	17
Parietal	6	4	/	10	20
Occipital	2	1	2	/	5
Temporal	4	3	/	6	13
Sphenoid	/	/	/	/	/
Incisive	/	/	/	/	/
Nasal	/	/	/	/	/
Lacrymal	/	/	/	/	/
Zygomatic	4	7	/	/	11
Palatine	/	/	/	/	/
Ethmoid	/	/	/	/	/
Mandible	6	4	1	0	11
Isolated Teeth	27	24	/	3	54
Mandible fragments with teeth	/	1	/	/	1
Maxilla fragments with teeth	/	/	/	/	/

Supplementary Table 11. Cercopithecid dental elements recovered from the Late Pleistocene layers (Phase D) of Fa-Hien Lena.

	Teeth (isolated and maxillary/mandibular)			
	Upper		Lower	
	Right	Left	Right	Left
Unid	3			
dc	/	/	/	/
dp3	/	/	/	/
dp4	/	/	1	/
I1	2	3	1	1
I2	1	2	2	1
C	3	2	3	1
P3	/	1	2	/
P4	2	1	/	1
M1	2	3	/	2
M2	3	1	1	2
M3	2	2	2	1

Supplementary Table 12. Cercopithecid pectoral and pelvic girdle elements and vertebrae recovered from the Late Pleistocene layers (Phase D) of Fa-Hien Lena.

		NISP			Total
		Right	Left	Indet.	
Patella		3	2	/	5
Scapula		7	4	4	15
Clavicle		2	1	/	3
Pelvis		7	10	/	17
	Ischium	9	8	/	
	Illium	6	10	/	
	Acetabulum	4	3	/	
	Pubis	3	2	/	
Vertebrae					31
	Cervical		2		
	Thoracic		2		
	Lumbar		3		
	Sacral		0		
	Caudal		24		

Supplementary Table 13. Cercopithecid hand and foot elements recovered from the Late Pleistocene layers (Phase D) of Fa-Hien Lena.

		NISP			Total
		Right	Left	Indet.	
Metacarpal	MC1	4	2	/	6
	MC2	4	3	/	7
	MC3	5	4	/	9
	MC4	3	3	/	6
	MC5	2	3	/	5
TOTAL					33
Carpal	Trapezium			/	
	Trapezoid	1	0	/	1
	Capitate	2	1	/	3
	Hamate	1	1	/	2
	Scaphoid	2	0	/	2
	Lunate	1	1	/	2
	Pyramidal	0	1	/	1
	Pisiform			/	
TOTAL					11
Metatarsal	MT1	4	3	/	7
	MT2	6	3	/	9
	MT3	5	4	/	9
	MT4	4	5	/	9
	MT5	4	1	/	5
TOTAL					39
Tarsals	Astragalus	1	1	/	2
	Calcaneus	1	3	/	4
	Cuboid	/	1	/	1
	Navicular	1	/	/	1
	Cuneiform	/	/	/	/
TOTAL					8
Metapodial Shaft Fragments					28
Phalanges					54

Supplementary Table 14. Primate long bone fragments recovered from the Late Pleistocene layers (Phase D) of Fa-Hien Lena.

	NISP									Total
	Proximal			Diaphyses			Distal			
	Right	Left	Indet	Right	Left	Indet	Right	Left	Indet	
Humerus	4	2	/	12	11	10	2	3	/	44
Ulna	4	1	/	5	6	2	1	1	/	20
Radius	2	1	/	7	4	5	1	2	/	22
Femur	4	2	/	31	20	16	4	5	/	82
Tibia	1	3	/	10	8	4	1	2	/	29
Fibula	1	/	/	7	8	2	/	1	/	19

Supplementary Table 15. Cercopithecid skull and mandible fragments recovered from the mid-Holocene layers (Phase A) of Fa-Hien Lena.

	NISP				Total
	Right	Left	R/L	Indet.	
Skull Fragments					10
Maxilla	/	2	/	/	2
Frontal	/	/	/	/	/
Parietal	1	/	/	3	4
Occipital	/	/	/	/	/
Temporal	1	/	/	1	2
Sphenoid	/	/	/	/	/
Incisive	/	/	/	/	/
Nasal	/	/	/	/	/
Lacrymal	/	/	/	/	/
Zygomatic	1	1	/	/	2
Palatine	/	/	/	/	/
Ethmoid	/	/	/	/	/
Mandible	3	3	/	/	6
Isolated Teeth	3	3	/	/	6
Mandible fragments with teeth	3	3	/	/	6
Maxilla fragments with teeth	/	1	/	/	1

Supplementary Table 16. Cercopithecid dental elements recovered from the mid-Holocene layers (Phase A) of Fa-Hien Lena.

	Teeth (isolated and maxillary/mandibular)			
	Upper		Lower	
	Right	Left	Right	Left
Unid		/		
dc	/	/	/	/
dp3	/	/	/	/
dp4	/	/	/	/
I1	/	/	1	/
I2	/	/	/	/
C	/	1	2	1
P3	/	/	/	/
P4	/	/	/	/
M1	/	1	/	/
M2	/	/	/	/
M3	/	/	/	/

Supplementary Table 17. Cercopithecid pectoral and pelvic girdle elements and vertebrae recovered from the mid-Holocene layers (Phase A) of Fa-Hien Lena.

		NISP			Total
		Right	Left	Indet.	
Patella		/	1	/	1
Scapula		7	5	3	15
Clavicle		1	/	/	1
Pelvis					19
	Ischium	6	4	/	
	Illium	3	4	2	
	Acetabulum	5	4	4	
	Pubis	2	3	1	
Vertebrae					18
	Cervical		3		
	Thoracic		2		
	Lumbar		2		
	Sacral		1		
	Caudal		10		

Supplementary Table 18. Cercopithecid hand and foot elements recovered from the mid-Holocene layers (Phase A) of Fa-Hien Lena.

		NISP			Total
		Right	Left	Indet.	
Metacarpal	MC1	/	/	/	/
	MC2	/	1	/	1
	MC3	2	1	/	3
	MC4	1	2	/	3
	MC5	1	/	/	1
TOTAL					8
Carpal	Trapezium	/	/	/	/
	Trapezoid	/	/	/	/
	Capitate	/	/	/	/
	Hamate	/	/	/	/
	Scaphoid	/	/	/	/
	Lunate	1	/	/	1
	Pyramidal	/	/	/	/
	Pisiform	/	/	/	/
TOTAL					1
Metatarsal	MT1	1	3	/	4
	MT2	3	/	/	3
	MT3	/	2	/	2
	MT4	1	/	/	1
	MT5	/	2	/	2
TOTAL					12
Tarsals	Astragalus	2	1	/	3
	Calcaneus	4	5	/	9
	Cuboid	1	/	/	1
	Navicular	/	/	/	/
	Cuneiform	/	/	/	/
TOTAL					26
Metapodial Shaft Fragments					6
Phalanges					4

Supplementary Table 19. Cercopithecid long bone fragments recovered from the mid-Holocene layers (Phase A) of Fa-Hien Lena.

	NISP									Total
	Proximal			Diaphyses			Distal			
	Right	Left	Indet	Right	Left	Indet	Right	Left	Indet	
Humerus	1	2	/	3	2	3	3	2	/	16
Ulna	18	13	/	10	12	/	1	2	/	56
Radius	2	1	/	2	3	6	4	3	1	22
Femur	2	4	/	6	4	13	3	2	/	34
Tibia	1	2	/	4	3	4	2	3	/	19
Fibula	2	1	/	6	4	2	3	1	/	19

Supplementary Table 20. Cercopithecid skull and mandible fragments recovered from the Early Holocene layers (Phase B) of Fa-Hien Lena.

	NISP				Total
	Right	Left	R/L	Indet.	
Skull Fragments					109
Maxilla	6	7	8	/	21
Frontal	4	2	/	3	9
Parietal	8	7	/	32	47
Occipital	2	1	1	1	5
Temporal	5	8	/	15	28
Sphenoid	/	/	/	/	/
Incisive	/	1	/	/	1
Nasal	1	/	/	/	1
Lacrymal	/	/	/	/	/
Zygomatic	3	2	/	/	5
Palatine	/	/	/	/	/
Ethmoid	/	/	/	/	/
Mandible	2	6	2	1	11
Isolated Teeth	24	35	/	4	65
Mandible fragments with teeth	/	3	1	/	4
Maxilla fragments with teeth	1	3	/	/	4

Supplementary Table 21. Cercopithecid dental elements recovered from the Early Holocene layers (Phase B) of Fa-Hien Lena.

	Teeth (isolated and maxillary/mandibular)			
	Upper		Lower	
	Right	Left	Right	Left
Unid		4		
dc	1	/	1	/
dp3	/	/	/	1
dp4	1	2	2	/
I1	1	/	1	/
I2	/	2	3	1
C	1	1	1	2
P3	1	2	1	3
P4	/	2	/	1
M1	4	7	2	2
M2	/	3	1	2
M3	1	1	4	3

Supplementary Table 22. Cercopithecid pectoral and pelvic girdle elements and vertebrae recovered from the Early Holocene layers (Phase B) of Fa-Hien Lena.

		NISP			Total
		Right	Left	Indet.	
Patella		2	1	/	3
Scapula		7	8	15	30
Clavicle		1	2	/	3
Pelvis					64
	Ischium	14	16	/	
	Illium	31	28	2	
	Acetabulum	18	21	/	
	Pubis	26	21	/	
Vertebrae					79
	Cervical		6		
	Thoracic		5		
	Lumbar		9		
	Sacral		2		
	Caudal		57		

Supplementary Table 23. Cercopithecid hand and foot elements recovered from the Early Holocene layers (Phase B) of Fa-Hien Lena.

		NISP			Total
		Right	Left	Indet.	
Metacarpal	MC1	4	3	/	7
	MC2	8	8	/	16
	MC3	8	9	/	17
	MC4	3	5	/	8
	MC5	1	3	/	4
TOTAL					48
Carpal	Trapezium	/	/	/	/
	Trapezoid	/	1	/	1
	Capitate	2	/	/	2
	Hamate	2	/	/	2
	Scaphoid	1	1	/	2
	Lunate	/	1	/	1
	Pyramidal	/	1	/	1
	Pisiform	/	/	/	/
TOTAL					9
Metatarsal	MT1	3	4	/	7
	MT2	9	6	/	15
	MT3	10	7	/	17
	MT4	7	11	/	18
	MT5	3	2	/	5
TOTAL					62
Tarsals	Astragalus	4	5	/	9
	Calcaneus	5	5	/	10
	Cuboid	4	1	/	5
	Navicular	1	1	/	2
	Cuneiform	2	2	/	4
TOTAL					30
Metapodial Shaft Fragments					80
Phalanges					149

Supplementary Table 24. Cercopithecid long bone fragments recovered from the Early Holocene layers (Phase B) of Fa-Hien Lena.

	NISP									Total
	Proximal			Diaphyses			Distal			
	Right	Left	Indet	Right	Left	Indet	Right	Left	Indet	
Humerus	4	3	/	15	13	28	6	8	/	77
Ulna	18	20	4	8	6	15	2	1	4	78
Radius	4	5	/	12	10	14	4	6	/	55
Femur	16	14	/	20	15	27	10	12	/	114
Tibia	4	5	/	18	16	6	4	2	/	55
Fibula	3	5	/	10	8	4	7	4	/	41

Supplementary Table 25. Cercopithecid skull and mandible fragments recovered from the Terminal Pleistocene layers (Phase C) of Fa-Hien Lena.

	NISP				Total
	Right	Left	R/L	Indet.	
Skull Fragments					264
Maxilla	9	19	/	8	36
Frontal	11	11	1	11	34
Parietal	24	30	3	26	83
Occipital	5	5	8	4	22
Temporal	23	19	20	21	83
Sphenoid	/	/	/	/	/
Incisive	/	/	/	/	/
Nasal	2	0	0	0	2
Lacrymal	/	/	/	/	/
Zygomatic	5	8	/	/	13
Palatine	2	1	1	2	6
Ethmoid	/	/	/	/	/
Mandible	15	29	9	7	60
Isolated Teeth	114	112	/	18	244
Mandible fragments with teeth	1	4	/	/	5
Maxilla fragments with teeth	/	2	/	/	2

Supplementary Table 26. Cercopithecid dental elements recovered from the Terminal Pleistocene layers (Phase C) of Fa-Hien Lena.

	Teeth (isolated and maxillary/mandibular)			
	Upper		Lower	
	Right	Left	Right	Left
Unid		18		
dc	2	/	1	1
dp3	1	2	1	/
dp4	4	2	2	1
I1	6	7	7	8
I2	7	4	4	6
C	8	10	8	7
P3	3	4	4	6
P4	7	3	3	1
M1	5	5	13	9
M2	3	5	11	14
M3	3	3	11	14

Supplementary Table 27. Cercopithecid pectoral and pelvic girdle elements and vertebrae recovered from the Terminal Pleistocene layers (Phase C) of Fa-Hien Lena.

		NISP			Total
		Right	Left	Indet.	
Patella		6	4	/	10
Scapula		34	27	3	64
Clavicle		4	6	/	10
Pelvis		29	30	/	59
	Ischium	14	7	/	
	Illium	20	21	/	
	Acetabulum	12	16	/	
	Pubis	8	12	/	
Vertebrae					238
	Cervical		28		
	Thoracic		36		
	Lumbar		37		
	Sacral		10		
	Caudal		127		

Supplementary Table 28. Cercopithecid hand and foot elements recovered from the Terminal Pleistocene layers (Phase C) of Fa-Hien Lena.

		NISP			Total
		Right	Left	Indet.	
Metacarpal	MC1	9	4	/	13
	MC2	17	12	/	29
	MC3	12	15	/	27
	MC4	6	5	/	11
	MC5	7	4	/	11
TOTAL					91
Carpal	Trapezium	1	/	/	1
	Trapezoid	/	1	/	1
	Capitate	2	2	/	4
	Hamate	/	1	/	1
	Scaphoid	/	2	/	2
	Lunate	1	/	/	1
	Pyramidal	1	1	/	2
	Pisiform	/	/	/	/
TOTAL					12
Metatarsal	MT1	5	6	/	11
	MT2	12	16	/	28
	MT3	17	13	/	30
	MT4	14	16	/	30
	MT5	7	9	/	16
TOTAL					115
Tarsals	Astragalus	17	11	/	28
	Calcaneus	18	21	/	39
	Cuboid	1	4	/	5
	Navicular	3	4	/	7
	Cuneiform	3	2	/	5
TOTAL					84
Metapodial Shaft Fragments					123
Phalanges					135

Supplementary Table 29. Cercopithecid long bone fragments recovered from the Terminal Pleistocene layers (Phase C) of Fa-Hien Lena.

	NISP									Total
	Proximal			Diaphyses			Distal			
	Right	Left	Indet	Right	Left	Indet	Right	Left	Indet	
Humerus	14	10	2	20	19	47	21	16	5	154
Ulna	19	14	8	20	18	13	2	1	9	104
Radius	9	8	/	15	14	13	7	5	/	71
Femur	20	25	/	23	15	55	12	5	6	161
Tibia	14	12	/	15	8	22	4	2	/	77
Fibula	2	3	/	20	13	20	3	1	/	62

Supplementary Table 30. Minimum Number of Element (MNE) and percent Minimum Animal Unit (%MAU) values for the cercopithecid remains recorded from the Late Pleistocene layers (Phase D) of Fa-Hien Lena.

		MNE	MAU	% MAU
Scapula		11	5.5	39.29
Clavicle		3	1.5	10.71
Humerus		10	5.5	39.29
Radius		8	4	28.57
Ulna		5	2.5	17.86
Metacarpal	1	6	3	21.43
	2	7	3.5	25
	3	9	4.5	32.14
	4	6	3	21.43
	5	5	2.5	17.86
Carpal	Trapezium	/	/	/
	Trapezoid	2	1	7.14
	Capitate	3	1.5	10.71
	Hamate	1	0.5	3.57
	Scaphoid	3	1.5	10.71
	Lunate	2	1	7.14
	Pyramidal	/	/	/
	Pisiform	/	/	/
Pelvis	Ilium	12	6	42.86
	Ischium	10	5	35.71
	Acetabulum	7	3.5	25
	Pubis	5	2.5	17.86
Femur		28	14	100
Patella		5	2.5	17.86
Tibia		12	6	42.86
Fibula		13	6.5	46.3
Metatarsal	1	7	3.5	25
	2	9	4.5	32.14
	3	9	4.5	32.14
	4	9	4.5	32.14
	5	5	2.5	17.86
Tarsal	Astragalus	2	1	7.14
	Calcaneus	4	2	14.29
	Cuboid	1	0.5	3.57
	Navicular	1	0.5	3.57
Phalanges	Proximal	31	1.55	11.07
	Intermediate	18	0.9	6.43

	Distal	5	0.25	1.79
Vertebra	Cervical	2	0.29	2.07
	Thoracic	2	0.17	1.21
	Lumbar	3	0.43	3.07
	Sacral	/	/	/
	Caudal	24	1.33	9.5
Mandible		7	3.5	25

Supplementary Table 31. Minimum Number of Element (MNE) and percent Minimum Animal Unit (%MAU) values for the cercopithecid remains recorded from the Terminal Pleistocene layers (Phase C) of Fa-Hien Lena.

		MNE	MAU	% MAU
Scapula		36	18	80
Clavicle		10	5	22.22
Humerus		37	18.5	82.22
Radius		17	8.5	37.78
Ulna		33	16.5	73.33
Metacarpal	1	13	6.5	28.89
	2	29	14.5	64.44
	3	27	13.5	60
	4	11	5.5	24.44
	5	11	5.5	24.44
Carpal	Trapezium	1	0.5	2.22
	Trapezoid	1	0.5	2.22
	Capitate	4	2	8.89
	Hamate	1	0.5	2.22
	Scaphoid	2	1	4.44
	Lunate	1	0.5	2.22
	Pyramidal	2	1	4.44
	Pisiform	/	/	/
Pelvis	Ilium	31	15.5	68.89
	Ischium	21	10.5	46.67
	Acetabulum	28	14	62.22
	Pubis	20	10	44.44
Femur		45	22.5	100
Patella		10	5	22.22
Tibia		26	13	57.78
Fibula		32	16	71.11
Metatarsal	1	11	5.5	24.44
	2	29	14.5	64.44
	3	30	15	66.67
	4	30	15	66.67
	5	16	8	35.56
Tarsal	Astragalus	28	14	62.22
	Calcaneus	39	19.5	86.67
	Cuboid	5	2.5	11.11
	Navicular	7	3.5	15.56

Phalanges	Proximal	78	3.9	17.33
	Intermediate	42	2.1	9.33
	Distal	15	0.75	3.33
Vertebra	Cervical	28	4	17.78
	Thoracic	36	3	13.33
	Lumbar	37	5.29	23.51
	Sacral	10	10	44.44
	Caudal	127	7.05	31.36
Mandible		34	17	75.56

Supplementary Table 32. Minimum Number of Element (MNE) and percent Minimum Animal Unit (%MAU) values for the cercopithecid remains recorded from the Early Holocene layers (Phase B) of Fa-Hien Lena.

		MNE	MAU	% MAU
Scapula		15	7.5	39.47
Clavicle		3	1.5	7.89
Humerus		14	7	36.84
Radius		10	5	26.32
Ulna		38	19	100
Metacarpal	1	7	3.5	18.42
	2	16	8	42.11
	3	17	8.5	44.74
	4	8	4	21.05
	5	4	2	10.53
Carpal	Trapezium	/	/	/
	Trapezoid	1	0.5	2.63
	Capitate	2	1	5.26
	Hamate	2	1	5.26
	Scaphoid	2	1	5.26
	Lunate	1	0.5	2.63
	Pyramidal	1	0.5	2.63
	Pisiform	/	/	/
Pelvis	Ilium	28	14	73.68
	Ischium	20	10	52.63
	Acetabulum	36	18	94.74
	Pubis	26	13	68.42
Femur		30	15	78.95
Patella		3	1.5	7.89
Tibia		9	4.5	23.68
Fibula		11	5.5	28.95
Metatarsal	1	7	3.5	18.42
	2	15	7.5	39.47
	3	17	8.5	44.74
	4	18	9	47.37
	5	5	2.5	13.16
Tarsal	Astragalus	9	4.5	23.68
	Calcaneus	10	5	26.32
	Cuboid	5	2.5	13.16
	Navicular	2	1	5.26
Phalanges	Proximal	89	4.45	23.42

	Intermediate	42	2.1	11.05
	Distal	18	0.9	4.74
Vertebra	Cervical	6	0.86	4.53
	Thoracic	5	0.42	2.21
	Lumbar	9	1.29	6.79
	Sacral	2	2	10.53
	Caudal	57	3.17	16.68
Mandible		12	6	31.58

Supplementary Table 33. Minimum Number of Element (MNE) and percent Minimum Animal Unit (%MAU) values for the cercopithecid remains recorded from the mid-Holocene layers (Phase A) of Fa-Hien Lena.

		MNE	MAU	% MAU
Scapula		12	6	38.71
Clavicle		1	0.5	3.23
Humerus		5	2.5	16.13
Radius		7	3.5	22.58
Ulna		31	15.5	100
Metacarpal	1	/	/	/
	2	1	0.5	3.23
	3	3	1.5	9.68
	4	3	1.5	9.68
	5	1	0.5	3.23
Carpal	Trapezium	/	/	/
	Trapezoid	/	/	/
	Capitate	/	/	/
	Hamate	/	/	/
	Scaphoid	/	/	/
	Lunate	1	0.5	3.23
	Pyramidal	/	/	/
	Pisiform	/	/	/
Pelvis	Ilium	7	3.5	22.58
	Ischium	10	5	32.26
	Acetabulum	9	4.5	29.03
	Pubis	5	2.5	16.13
Femur		6	3	19.35
Patella		1	0.5	3.23
Tibia		5	2.5	16.13
Fibula		4	2	12.9
Metatarsal	1	4	2	12.9
	2	3	1.5	9.68
	3	2	1	6.45
	4	1	0.5	3.23
	5	2	1	6.45
Tarsal	Astragalus	3	1.5	9.68
	Calcaneus	9	4.5	29.03
	Cuboid	1	0.5	3.23
	Navicular	/	/	/
Phalanges	Proximal	2	0.1	0.65
	Intermediate	2	0.1	0.65

	Distal	/	/	/
Vertebra	Cervical	3	0.43	2.77
	Thoracic	2	0.17	1.1
	Lumbar	2	0.29	1.87
	Sacral	1	1	6.45
	Caudal	10	0.56	3.61
Mandible		6	3	19.35

Supplementary Table 34. Cercopithecid long bone fragmentation (NISP:MNE) and fragment completeness.

Phase	Element	NISP:MNE Ratio	Circumferential completeness			Length completeness		
			100%	> 50%, <100%	<50%	100%	> 50%, <100%	<50%
Phase A	Humerus	3.2	37.5	37.5	25	0	6.25	93.75
	Ulna	1.81	67.86	25	7.14	0	26.79	73.21
	Radius	3.14	77.27	9.09	13.64	0	50	50
	Femur	5.67	76.47	14.71	8.82	0	14.71	85.29
	Tibia	3.8	78.95	10.53	10.53	0	26.32	73.68
	Fibula	4.75	57.89	42.11	0	5.26	26.32	68.42
Phase B								
	Humerus	5.5	24.68	28.57	46.75	0	18.18	81.82
	Ulna	2.05	71.79	20.51	7.69	0	24.36	75.64
	Radius	5.5	61.82	29.09	9.09	0	18.18	81.82
	Femur	3.8	8.77	25.44	65.79	0	29.82	70.18
	Tibia	6.11	36.36	25.45	38.18	0	36.36	63.64
	Fibula	3.73	75.61	24.39	0	2.44	26.83	70.73
Phase C								
	Humerus	4.16	62.99	10.39	26.62	0	26.62	73.38
	Ulna	3.15	69.23	20.19	10.58	0	50.96	49.04
	Radius	4.18	45.07	38.03	16.9	0	25.35	74.65
	Femur	3.58	22.98	25.47	51.55	0	19.88	80.12
	Tibia	2.96	38.96	35.06	25.97	0	38.96	61.04
	Fibula	1.94	72.58	24.19	3.23	0	19.48	75.81
Phase D								
	Humerus	4.4	9.09	15.91	75	0	36.36	63.64
	Ulna	4	60	30	10	0	25	75
	Radius	2.75	59.09	22.73	18.18	0	18.18	81.82
	Femur	2.93	4.88	8.54	86.59	0	24.39	75.61
	Tibia	2.42	6.9	27.59	65.52	0	13.79	86.21
	Fibula	1.46	57.89	36.84	5.26	0	47.37	52.63

Supplementary Table 35. Anthropic modifications in bone fragments recovered from Fa-Hien Lena.

Phase	Context	TNF	NISP	Burning				Butchery	Tools/Ornaments
				Partial	Total	PC	TC		
A	216	661	362	3	29	22	25	9	4
	131	114	53	6	7	22	7	2	2
	135	181	74	1	3	2	1	2	10
B	7/151	62	39	3	7	15	9	0	2
	52/153	451	331	24	12	48	32	1	1
	170	188	84	16	23	15	14	3	10
	116/128	2281	838	45	133	39	20	2	18
	180	380	165	22	15	26	23	1	5
	136	535	256	32	70	85	23	6	7
	38/206	193	66	2	12	23	24	1	3
	138	210	161	3	11	3	5	1	4
	98	35	13	1	2	3	1	0	0
	107	260	260	3	4	1	1	0	1
C	168	87	42	4	13	11	6	3	2
	237	93	71	3	5	2	3	1	4
	139/140	576	257	29	59	25	7	14	9
	141	94	78	3	5	2	1	0	5
	173	605	293	105	185	78	121	1	3
	142	207	41	1	2	0	0	1	2
	248	2354	1512	84	147	39	28	24	59
	174/247	2294	1161	66	113	73	67	11	53
	144/161/164	245	196	8	13	2	1	0	3
	163/235	60	60	3	4	1	1	0	4
D	108	318	237	0	17	19	2	1	1
	110	414	267	12	15	17	7	0	2
	118	238	86	13	41	34	14	2	1
	145	36	29	0	1	0	1	1	1
	179	132	90	2	2	19	28	1	1
	175	288	143	12	36	19	10	1	15
	157	55	26	3	6	7	4	0	2
	158	64	34	0	5	1	1	0	1
	159	31	12	2	2	0	1	0	1
	165	38	12	0	4	1	1	0	1
	253	705	280	29	71	62	33	3	10

Supplementary Table 36. Minimum Number of Element (MNE) and percent Minimum Animal Unit (%MAU) values for the sciurid remains recorded from the mid-Holocene layers (Phase A) of Fa-Hien Lena.

		NISP			MNE	MAU	% MAU
		Right	Left	Indet			
Scapula		/	1	/	1	0.5	20
Humerus	Proximal	1	/	/	3	1.5	60
	Shaft	2	1	/			
	Distal	/	2	/			
Radius	Proximal	1	/	/	3	1.5	60
	Shaft	1	2	/			
	Distal	/	/	/			
Ulna	Proximal	3	2	/	5	2.5	100
	Shaft	1	1	/			
	Distal	/	/	/			
Metacarpal	1	/	/	/	/	/	/
	2	1	/	/	1	0.5	20
	3	/	1	/	1	0.5	20
	4	1	/	/	1	0.5	20
	5	/	/	/	/	/	/
Carpal	Trapezium	/	/	/	/	/	/
	Trapezoid	/	/	/	/	/	/
	Capitate	/	/	/	/	/	/
	Hamate	/	/	/	/	/	/
	Scaphoid	/	/	/	/	/	/
	Lunate	/	/	/	/	/	/
	Pyramidal	/	/	/	/	/	/
	Pisiform	/	/	/	/	/	/
Pelvis	Ilium	2	1	/	3	1.5	60
	Ischium	1	3	/			
	Acetabulum	1	3	/			
	Pubis	1	1	/			
Femur	Proximal	2	1	/	3	1.5	60
	Shaft	1	1	/			
	Distal	1	1	/			
Tibia	Proximal	2	/	/	2	1	40
	Shaft	1	1	/			
	Distal	/	1	/			
Metatarsal	1	/	/	/	/	/	/
	2	/	/	/	/	/	/
	3	1	/	/	1	0.5	20
	4	/	1	/	1	0.5	20
	5	/	/	/	/	/	/
Tarsal	Astragalus	1	/	/	1	0.5	20
	Calcaneus	/	1	/	1	0.5	20
Phalanges	Proximal		1		1	0.05	2
	Intermediate		2		2	0.1	4
	Distal		/		/	/	/
Vertebra	Cervical		/		/	/	/

Thoracic		1		/	0.08	3.2
Lumbar		/		/	/	/
Sacral		/		/	/	/
Caudal		4		/	0.13	5.2
Mandible	1	2	/	3	1.5	60

Supplementary Table 37. Minimum Number of Element (MNE) and percent Minimum Animal Unit (%MAU) values for the sciurid remains recorded from the Early Holocene layers (Phase B) of Fa-Hien Lena.

		NISP			MNE	MAU	% MAU
		Right	Left	Indet			
Scapula		3	4	/	7	3.5	16.3
Humerus	Proximal	19	23	/	42	21	97.7
	Shaft	24	23	/			
	Distal	14	18	/			
Radius	Proximal	16	15	/	31	15.5	72.1
	Shaft	12	10	/			
	Distal	1	2	/			
Ulna	Proximal	21	22	/	43	21.5	100
	Shaft	26	26	/			
	Distal	1	2	/			
Metacarpal	1	/	/	/	/	/	/
	2	1	2	/	3	1.5	7
	3	2	1	/	3	1.5	7
	4	1	/	/	1	0.5	2.3
	5	/	/	/	/	/	/
Carpal	Trapezium	1	/	/	1	0.5	2.3
	Trapezoid	/	/	/	/	/	/
	Capitate	/	/	/	/	/	/
	Hamate	/	/	/	/	/	/
	Scaphoid	/	1	/	1	0.5	2.3
	Lunate	/	/	/	/	/	/
	Pyramidal	/	/	/	/	/	/
	Pisiform	/	/	/	/	/	/
Pelvis	Ilium	11	12	/	31	15.5	72.1
	Ischium	18	14	/			
	Acetabulum	21	19	/			
	Pubis	14	17	/			
Femur	Proximal	21	22	/	43	21.5	100
	Shaft	19	21	/			
	Distal	4	5	/			
Patella		1	/	/	1	0.5	2.3
Tibia	Proximal	14	16	/	30	15	69.8
	Shaft	15	11	/			
	Distal	4	6	/			
Metatarsal	1	/	/	/	/	/	/
	2	1	3	/	4	2	9.3
	3	1	2	/	3	1.5	7
	4	2	2	/	4	2	9.3
	5	1	/	/	4	2	9.3
Tarsal	Astragalus	1	1	/	2	1	4.7
	Calcaneus	12	8	/	20	10	46.5
Phalanges	Proximal		24		24	1.2	5.6
	Intermediate		28		28	1.4	6.5
	Distal		2		2	0.1	0.5

Vertebra	Cervical		7		7	1	4.7
	Thoracic		14		4	0.93	4.3
	Lumbar		11		11	1.83	8.5
	Sacral		2		2	2	9.3
	Caudal		34		34	1.13	5.3
Mandible		4	3	/	7	3.5	16.3

Supplementary Table 38. Minimum Number of Element (MNE) and percent Minimum Animal Unit (%MAU) values for the sciurid remains recorded from the Terminal Pleistocene layers (Phase C) of Fa-Hien Lena.

		NISP			MNE	MAU	% MAU
		Right	Left	Indet			
Scapula		14	16	/	30	15	51.7
Humerus	Proximal	8	7	/	51	25.5	87.9
	Shaft	24	36	10			
	Distal	11	8	/			
Radius	Proximal	4	1	/	21	10.5	36.2
	Shaft	8	16	8			
	Distal	3	4	/			
Ulna	Proximal	18	24	/	42	21	72.4
	Shaft	28	21	/			
	Distal	1	1	/			
Metacarpal	1	1	2	/	3	1.5	5.2
	2	4	2	/	6	3	10.3
	3	3	4	/	7	3.5	12.1
	4	2	4	/	6	3	10.3
	5	1	1	/	2	1	3.5
Carpal	Trapezium	1	/	/	1	0.5	1.7
	Trapezoid	/	/	/	/	/	/
	Capitate	1	1	/	2	1	3.5
	Hamate	1	/	/	1	0.5	1.7
	Scaphoid	/	/	/	/	/	/
	Lunate	/	1	/	1	0.5	1.7
	Pyramidal	1	/	/	1	0.5	1.7
	Pisiform	/	/	/	/	/	/
Pelvis	Ilium	24	21	/	41	20.5	70.7
	Ischium	14	11	/			
	Acetabulum	28	34	/			
	Pubis	8	11	/			
Femur	Proximal	17	22	/	58	29	100
	Shaft	44	35	28			
	Distal	22	21	/			
Patella		1	2	/	3	1.5	5.2
Tibia	Proximal	14	11	/	37	18.5	63.8
	Shaft	21	19	7			
	Distal	13	17	/			
Metatarsal	1	1	1	/	2	1	3.5
	2	1	2	/	3	1.5	5.2
	3	8	6	/	14	7	24.1
	4	7	4	/	11	5.5	18.9
	5	2	1	/	3	1.5	5.2
Tarsal	Astragalus	3	4	/	7	4.5	15.5
	Calcaneus	18	21	/	39	19.5	67.2
Phalanges	Proximal		48		48	2.4	8.3
	Intermediate		52		52	2.6	9
	Distal		14		14	0.7	2.4

Vertebra	Cervical		14		14	2	6.9
	Thoracic		8		8	0.6	2.1
	Lumbar		7		7	1.2	4.1
	Sacral		5		4	4	13.8
	Caudal		87		87	2.9	10
Mandible		21	31	/	48	24	82.8

Supplementary Table 39. Minimum Number of Element (MNE) and percent Minimum Animal Unit (%MAU) values for the sciurid remains recorded from the Late Pleistocene layers (Phase D) of Fa-Hien Lena.

		NISP			MNE	MAU	% MAU
		Right	Left	Indet			
Scapula		2	4	/	6	3	15
Humerus	Proximal	4	4	/	12	6	30
	Shaft	7	8	/			
	Distal	2	5	/			
Radius	Proximal	2	1	/	9	4.5	22.5
	Shaft	9	4	/			
	Distal	1	1	/			
Ulna	Proximal	10	8	/	18	9	45
	Shaft	9	12	/			
	Distal	/	/	2			
Metacarpal	1	1	/	/	1	0.5	2.5
	2	/	2	/	2	1	5
	3	1	2	/	3	1.5	7.5
	4	1	1	/	2	1	5
	5	1	/	/	1	0.5	2.5
Carpal	Trapezium	/	/	/	/	/	/
	Trapezoid	/	/	/	/	/	/
	Capitate	/	/	/	/	/	/
	Hamate	/	/	/	/	/	/
	Scaphoid	/	/	/	/	/	/
	Lunate	/	/	/	/	/	/
	Pyramidal	/	/	/	/	/	/
	Pisiform	/	/	/	/	/	/
Pelvis	Ilium	8	7	/	11	5.5	27.5
	Ischium	4	4	/			/
	Acetabulum	7	4	/			/
	Pubis	3	4	/			/
Femur	Proximal	4	8	/	40	20	100
	Shaft	24	20	/			
	Distal	8	7	/			
Patella		1	1	/	2	1	5
Tibia	Proximal	4	5	/	17	8.5	42.5
	Shaft	8	10	/			
	Distal	8	6	/			
Metatarsal	1	/	/	/	/	/	/
	2	1	/	/	1	0.5	2.5
	3	/	1	/	1	0.5	2.5

	4	2	1	/	3	1.5	7.5
	5	/	/	/	/	/	/
Tarsal	Astragalus	1	3	/	4	2	10
	Calcaneus	3	3	/	6	3	15
Phalanges	Proximal		12		12	0.6	3
	Intermediate		18		18	0.9	4.5
	Distal		4		4	0.2	1
Vertebra	Cervical		8		8	1.14	20
	Thoracic		6		6	0.46	15
	Lumbar		4		4	0.67	10
	Sacral		2		2	2	10
	Caudal		28		28	0.93	4.7
Mandible		6	4	/	10	5	25

Supplementary Table 40. Results of Kruskal-Wallis test and post-hoc pairwise comparison of mammalian taxa grouped by bod\y size in different occupational phases the site considering the NISP[29].

Kruskal-Wallis Test	H= 1.543, p= 0.6705, no significance between sample medians	
	Mann-Whitney Test with Bonferroni sequential correction for p	Dunn's Test with Bonferroni sequential correction for p
Phase A vs. B	0.5993	0.5201
Phase A vs. C	0.2984	0.2177
Phase A vs. D	0.6733	0.6296
Phase B vs. C	0.6004	0.5555
Phase B vs. D	0.9166	0.8723
Phase C vs. D	0.5309	0.4531

Supplementary Table 41. Results of Kruskal-Wallis test and post-hoc pairwise comparison of mammalian taxa grouped by body size in different occupational phases the site considering the MNI[29].

Kruskal-Wallis Test	H= 1.324, p= 0.7235, no significance between sample medians	
	Mann-Whitney Test with Bonferroni sequential correction for p	Dunn's Test with Bonferroni sequential correction for p
Phase A vs. B	0.4005	0.402
Phase A vs. C	0.4005	0.2818
Phase A vs. D	0.7518	0.6473
Phase B vs. C	0.7518	0.8086
Phase B vs. D	0.7533	0.7064
Phase C vs. D	0.6742	0.536

Supplementary Table 42. Result of t test and descriptive statistics for the difference in burning in Cercopithecid and Sciurid specimens across different layers[29].

No. of burnt/calcined specimens	Cercopithecids			Squirids			95% CI for Mean difference	t	df
	M	SD	n	M	SD	n			
	19.882	19.392	34	19.471	18.094	34	-8.6694, 9.493	0.0905	66

Supplementary Note 3. Lithic Materials

The use of bipolar technology was identified through diacritic reading of scar patterns on flakes and cores. This method involves holding the core on an anvil and striking it with a hard hammer. During the knapping process, force is transmitted on the vertical or tangential axis of the core and flakes are produced by the contact with the stone hammer and the anvil[30]. This results in the recurrent presence of bidirectional scars on both cores and flake dorsal surfaces, alongside crushing of elements in contact with either the hammer or anvil. (Supplementary Figure 17). At Fa-Hien Lena, the bipolar method was used consistently throughout the excavated assemblages. Generally, quartz nodules were struck along the longest axis in order to maximize the size of the byproducts. However, in some examples, the prehistoric knappers changed the striking platform during reduction rotating the core by 90 degrees and exploiting the smaller surface. This technical expedient permits a better exploitation of irregular pebbles and produces flakes with orthogonal scars on the dorsal surface (Supplementary Figure 17 n° 9, 10, 11). At the site, the aim of the knapping activities is the production of small flakes and the presence of small blades and bladelets could be considered as an unintentional outcome rather than a planned strategy. In fact, in the lithic collection, laminar cores and byproducts for maintaining the convexities in the laminar reduction are absent.

The flake assemblage is mainly composed of fragments and little chips, whereas complete blanks are less numerous (Supplementary Tables 43-44). This is very common in contexts of quartz bipolar reduction due to the simple organization of the core and the absence of predetermination on the byproducts. Furthermore, bipolar technology typically involves more limited control on the use of force in contrast to other technologies, with a bending stress applied resulting in cracking of the platform, creation of large numbers of small chips, or fracturing flakes along the percussion axis (sirét). Phase D exhibits the highest proportion of siret (longitudinal fractures) fragments (14.5%), followed by Phase B (13.7%), Phase A (12.6%) and Phase C (10.2%). Analysis of the complete flakes indicates their varying occurrence throughout the excavated sequence, with higher frequencies recorded in Phase D (Supplementary Table 43-44). Except for some outliers, the comparison between the median values of the length of unbroken artefacts shows minimal differences between the phases of occupation (Supplementary Figure 18).

Retouched tools occur in extremely low frequency, numbering only three microliths discovered in Phase D (Supplementary Tables 43-44). The microlith is a small stone tool made on a bladelet or a small flake, characterized by a steep and blunting retouch along one edge[31]. The microlith could be laminar, if the blank has an elongated shape, or geometric, if the retouch changes the morphology of the blank in trapezoidal, triangular or crescent shape. Ethnographic data on modern-hunter gatherers and experimental studies indicate that microliths are commonly used hafted in composite tools or projectiles, and employed as hunting weapons[32-34].

A crescent microlith with a continuous backed retouch along one side and two notch fractures on the cutting edge was recorded in context 165 (Supplementary Figure 19A). A backed retouch on both edges were documented, respectively, in context 158 on a laminar microlith fragment with a bend fracture, and in context 175 on a laminar microlith with a step terminating breakage (Supplementary Figure 19B-C). Patterns of breakage on the three microliths are currently under study, but are likely to have originated from impacts occurring during hunting activities.

The analysis of the lithic materials from Fa-Hien Lena indicates a continuity in the use of bipolar knapping on anvil from the Late Pleistocene to the late Holocene. This method is very flexible and allows a greater exploitation of the nodule, in comparison with other technologies, producing relatively large blanks from small cores[34]. Moreover, the simple operative scheme enables the knapper to produce small blanks that are mostly straight and lacking pronounced bulbs on the ventral surfaces. These features make the bipolar flakes/blades particularly appropriate to be hafted after a minimal reshaping of the cutting edges. Similar technological strategies to those identified at Fa-Hien Lena have also been documented at Batadomba Lena and Kitulgala Beli-Lena, supporting the widespread diffusion of this complex behavior in the wet zone of Sri Lanka[36,37].

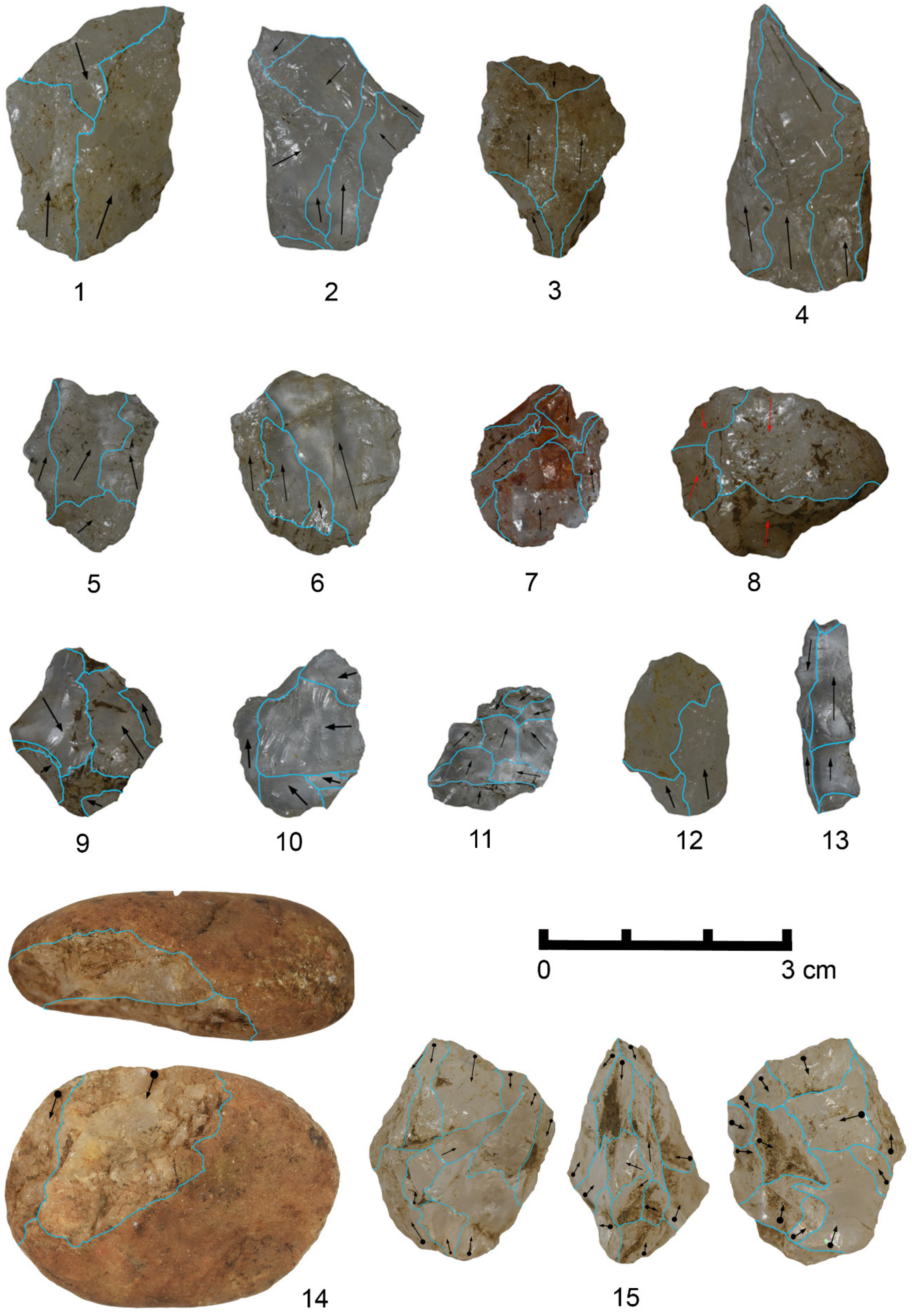

Supplementary Figure 17. Bipolar flakes from Phase D (1, 2, 4, 6, 10, 11), Phase C (3, 7, 9, 13), Phase B (5, 12) and bipolar cores from Phase D (15) and Phase C (14).

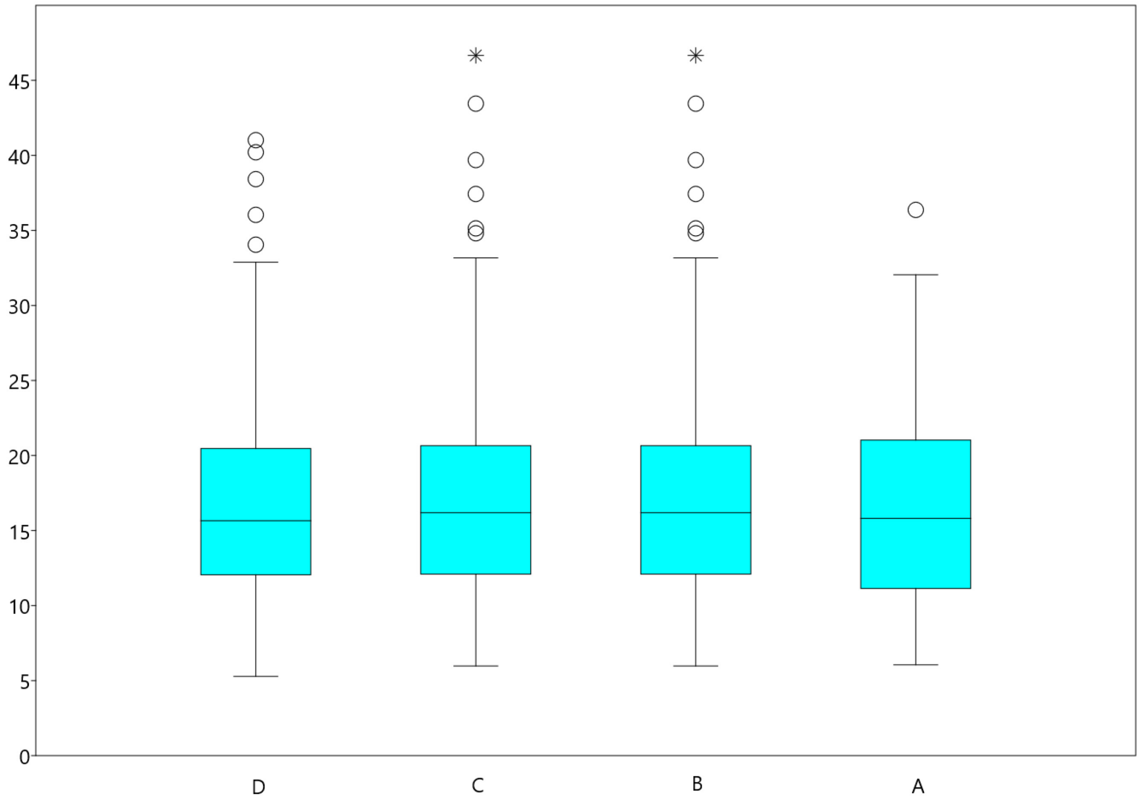

Supplementary Figure 18. Comparison of the length of complete flakes and blades in the different phases of occupation of Fa-Hien Lena Cave.

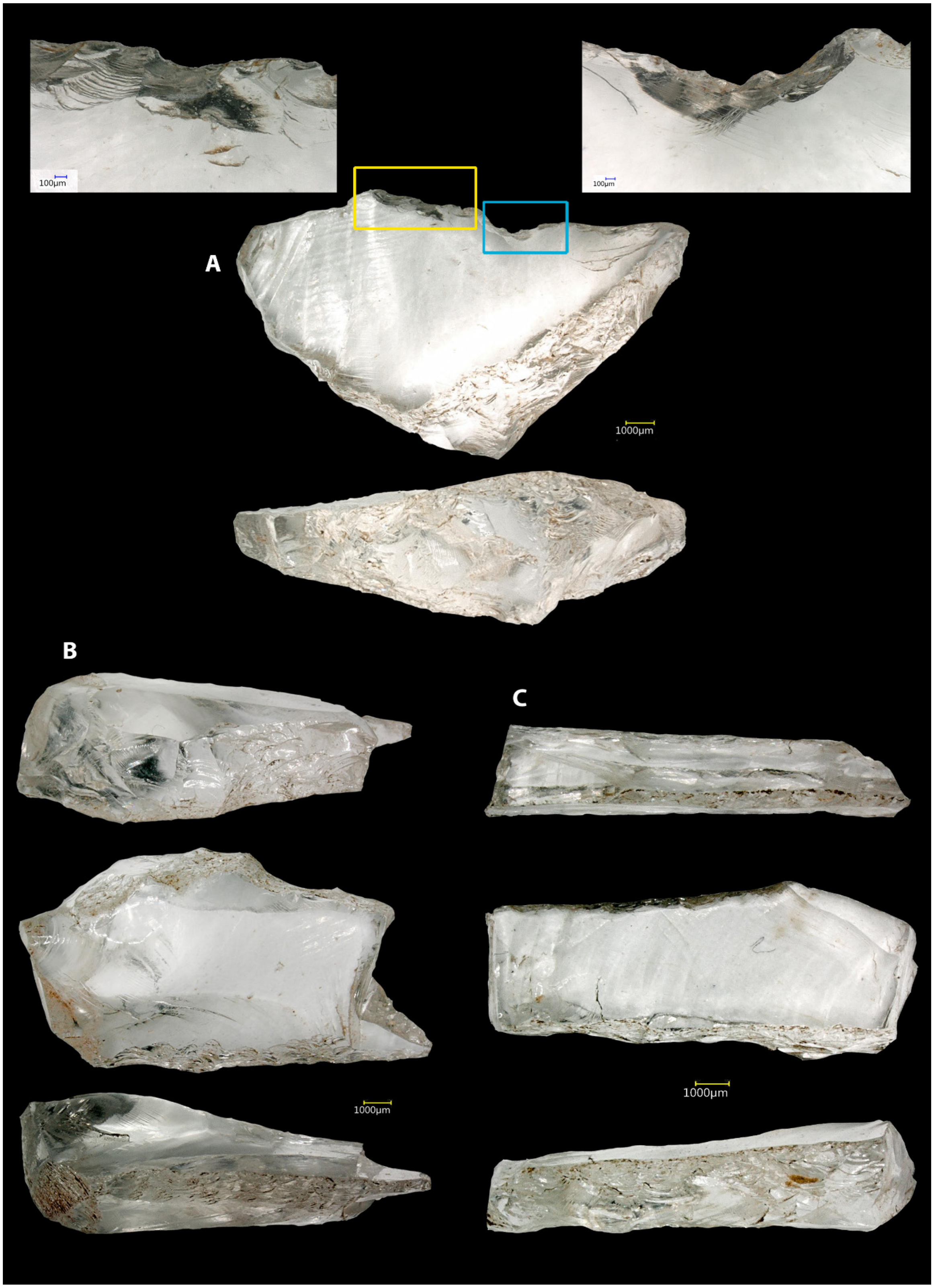

Supplementary Figure 19. Microlithic tools of Phase D context 165 (A), context 158 (B) and context 175 (C).

Supplementary Table 43. Total amount by contexts of the lithic materials of Fa-Hien Lena Cave.

	Layer	Flake	Blade	Fragments	Debris	Tools	Core	Core Frag.	Hammer	Total
A	216	8		14	19		1	2		44
	131	71	5	200	299		7	6		588
	31=32=135	19		61	28		3	1		112
B	7 = 151	11		25	14			2		52
	52=153	6		19	43		1	1		70
	170	7		7	5		2	2		23
	116=128	71	4	287	254		8	4		628
	136	42	1	73	71			1		188
	38=206	15		31	31					77
	138	10		27	52		3			92
	98	1		3	1					5
	107				7					7
C	168	9		23	67					99
	237	10		47	84		3	1		145
	139=140	30	1	140	361		4	4	1	541
	141	12		81	207		2		1	303
	173	7		26	46					79
	142	14		14	89		1			118
	248	6	3	13	6				1	29
	174 =247	75	1	212	352		7	5		652
	144=161=164	23	1	45	104			1		174
	163=235	5		9	32					46
D	108	10		31	38			1		80
	110	3		8			2			13
	118	11	3	33	5					52
	145	33	3	83	99		3			221
	179	20		15	43					78
	175	16	1	49	77	1		2	4	150
	157	11		10	17					38
	158	28	1	33	2	1	1	3		69
	159	28	3	86	3			3		123
	165	19	2	26	1	1				49
	253	25	1	65	32		2			125
**Total**		**656**	**30**	**1796**	**2489**	**3**	**50**	**39**	**7**	**5070**

Supplementary Table 44. Total amount of lithic materials in the different occupational phases of Fa-Hien Lena Cave.

Phase	Flake	Blade	Fragments	Debris	Tools	Core	Core Frag.	Hammer	Total
**A**	98	5	275	346		11	9		744
**%**	*13.2*	*0.7*	*37*	*46.5*		*1.5*	*1.2*		*100*
**B**	163	5	472	478		14	10		1142
**%**	*14.3*	*0.4*	*41.3*	*41.9*		*1.2*	*0.9*		*100*
**C**	191	6	610	1348		17	11	3	2186
**%**	*8.7*	*0.3*	*27.9*	*61.7*		*0.8*	*0.5*	*0.1*	*100*
**D**	204	14	439	317	3	8	9	4	998
**%**	*20.4*	*1.4*	*44*	*31.8*	*0.3*	*0.8*	*0.9*	*0.4*	*100*
**Total**	**656**	**30**	**1796**	**2489**	**3**	**50**	**39**	**7**	**5070**
**%**	***12.9***	***0.6***	***35.4***	***49.1***	***0.1***	***1***	***0.8***	***0.1***	***100***

Supplementary References

1 Stephens, M., Rose, J., Gilbertson, D., Canti, M. G. Micromorphology of cave sediments in the humid tropics: Niah Cave, Sarawak. *Asian Perspect.* **44**, 42–55 (2005).

2 Stephens, M., Roberts, R. G., Lian, O. B. & Yoshida, H. Progress in optical dating of guano-rich sediments associated with the Deep Skull, West Mouth of the Great Cave of Niah, Sarawak, Borneo. *Quat. Geochronol.* **2**, 330-336 (2007).

3 Perera, N. *et al.* People of the ancient rainforest: Late Pleistocene foragers at the Batadomba-lena rockshelter. *J. Hum. Evol.* **61,** 254-269 (2011).

4 Kennedy, K. A. R. & Elgart, A. A. Hominid Remains an Update. In *Supplement to Anthropologie et Prehistoire* (Eds. Orban, R. & Semal, P.) (Belgian Institute of Natural Sciences, 1998).

5 Perera, N. *Prehistoric Sri Lanka: Late Pleistocene Rockshelters and an Open Air Site.* (British Archaeological Reports International Series, Archaeopress, Oxford, 2010).

6 OxCal version 4.3 (2017).

7 Reimer, P. J. *et al*. IntCal13 and Marine13 radiocarbon age calibration curves 0–50,000 years cal BP. *Radiocarbon* **55**, 1869-188 (2013).

8 Gantt, D. G. Patterns of dental wear and the role of the canine in Cercopithecinae. *Am. J. Phys. Anthropol.* **51**, 353-359 (1979).

9 Ingicco, T., Moigne, A.-M. & Gommery, D. A deciduous and permanent dental wear stage system for assessing the age of *Trachypithecus* sp. specimens (Colobinae, Primates). *J. Archaeol. Sci.* **39,** 421-427 (2012).

10 Delson, E. *Fossil Colobine Monkeys of the Circum-Mediterranean Region and the Evolutionary History of the Cercopithecidae (Primates, Mammalia).* Ph.D. Dissertation Columbia University, New York, USA (1973).

11 Kay, R. F. The functional adaptations of primate molar teeth. *Am. J. Phys. Anthropol.* **43**, 195-216 (1975).

12 Kay, R. F. & Hylander, W. L. The dental structure of mammalian folivores with special reference to Primates and Phalangeroidea (Marsupialia). In *The Ecology of Arboreal Folivores* (Ed. Montgomery, G.) pp. 173-191 (Smithsonian Institution Press, Washington DC, 1978).

13 Lucas, P. W. & Teaford, M. F. Functional morphology of colobine teeth. In *Colobine Monkeys: Their Ecology, Behaviour and Evolution* (Eds. Davies, G. & Oates, J.) pp. 173-203 (Cambridge University Press, Cambridge, UK, 1994).

14 Swindler, D. R. *Dentition of Living Primates* (Academic Press, London, UK, 1976).

15 Swindler, D. R. *Primate Dentition: An Introduction to the Teeth of Non-Human Primates* (Cambridge University Press, Cambridge, UK, 2002).

16 Pan, R. L Dental variation among Asian colobines with special reference to the macaques on the same continent. *Zool. Res.* **28**, 569- 579 (2007).

17 Ky-Kidd, K. & Piper, P. J. Identification of morphological variation in the humeri of Bornean primates and its application to zooarchaeology. *Archaeofauna* **13**, 85-95 (2004).

18 Gebo, D. L. *Primate Comparative Anatomy* (Johns Hopkins Univ. Press. Baltimore, MD, 2014).

19 Nahallage, C. Craniometric analysis of two primate species from Sri Lanka: *Macaca sinica* and *Trachypithecus vetulus*. *Man and Environment* **40**, 27-32 (2015).

20 Piper, P. J. & Rabett, R. Late Pleistocene subsistence strategies in Island Southeast Asia and their implications for understanding the development of modern human behavior. In

Southern Asia, Australia, and the Search for Human Origins (Ed. Dennell, R.) pp. 118-134 (Cambridge Univ. Press, Cambridge, UK, 2004).
21 Piper, P. J., Rabett, R. E. & Kurui, B. Using community, composition and structural variation in terminal Pleistocene vertebrate assemblages to identify human hunting behaviour at the Niah Caves, Borneo. *Bulletin of the Indo-Pacific Prehistory Association* **28**, 88-98 (2008).
22 Barton, H., Rabett, R. & Piper, P. J. Composite hunting technologies from the Terminal Pleistocene and Early Holocene, Niah Cave, Borneo. *J. Archaeol. Sci.* **36**, 1708-1714.
23 Amano, N. Late Pleistocene to Mid-Holocene paleoenvironment and human subsistence strategies in Eastern Java (Indonesia). PhD Dissertation, *Muséum national d'Histoire naturelle*, Paris, France (2017).
24 Lyman, R. L. *Vertebrate Taphonomy*. (Cambridge Manuals in Archaeology, Cambridge University Press, Cambridge, UK, 1994).
25 Ingicco, T. Les primates quaternaires de SongTerus (Java Est, Indonésie) implications paléobiogéographiques et archéozoologiques pour l'Asie du Sud- Est. PhD Dissertation, *Muséum national d'Histoire naturelle*, Paris, France (2010).
26 Rabett, R. *Human Adaptation in the Asian Palaeolithic: Hominin Dispersal and Behaviour during the Late Quaternary* (Cambridge Univ. Press, Cambridge, UK, 2012).
27 Willis, L. M., Eren, M. I. & Rick, T. C. Does butchering fish leave cut marks? *J. Archaeol. Sci.* **35**, 1438-1444 (2008).
28 Willis, L. M. & Boehm, A. R. Fish bones, cut marks, and burial: implications for taphonomy and faunal analysis. *Journal of Archaeological Science* **45**, 20-25 (2014).
29 R Core Team (2018). R: A language and environment for statistical computing. R Foundation for Statistical Computing, Vienna, Austria. URL https://www.R-project.org/.
30 Crabtree, D. E. An Introduction to Flintworking. (Occasional Papers of the Idaho University Museum, No. 28, 1972).
31 Brézillon, M. N. *La Dénomination des Objets de Pierre Taillée. Matériaux pour un Vocabulaire des Préhistoriens de Langue Française* (Edition du Centre National de la Recherche Scientifique, Paris, France, 1968).
32 McCarthy, F.D. *Australian Aboriginal Stone Implements* (Australian Museum, Sydney, 1967).
33 Goldstein, S. T. & Shaffer, C. M. Experimental and archaeological investigations of backed microlith function among Mid-to-Late Holocene herders in southwestern Kenya. *Archaeol. Anthropol. Sci.* **9**, 1767-1788 (2017).
34 Lombard, M. & Pargeter, J. Hunting with Howiesons Poort segments: pilot experimental study and the functional interpretation of archaeological tools *J. Archaeol. Sci.* **35**, 2523-2531 (2008).
35 Hiscock, P. Making it small in the Palaeolithic: bipolar stone-working, miniature artefacts and models of core recycling. *World Archaeol.* **47**, 158-169 (2015).
36 Roberts, P., Boivin, N. & Petraglia, M. D. The Sri Lankan 'Microlithic' tradition c. 38,000 to 3000 years ago: Tropical technologies and adaptations of *Homo sapiens* at the southern edge of Asia. *J. World Prehist.* **28,** 69-112 (2015).
37 Lewis, L., Perera, N. & Petraglia, M. First technological comparison of Southern African Howiesons Poort and South Asian Microlithic industries: an exploration of inter-regional variability in microlithic assemblages. *Quat. Int.* **350**, 7-25 (2014).
