# Supplementary material for: Specialized rainforest hunting by Homo sapiens ~45,000 years ago
Source: Nat Commun. 2019 Feb 19;10:739. doi: 10.1038/s41467-019-08623-1 (PMC6381157; doi:10.1038/s41467-019-08623-1)
Supplement: Supplementary file 3 — Reporting Summary [file 41467_2019_8623_MOESM3_ESM.pdf]

nature research

Corresponding author(s): Oshan Wedage, Noel Amano, Michael Petraglia, Patrick Roberts

Last updated by author(s): 18.01.2019

Reporting Summary

Nature Research wishes to improve the reproducibility of the work that we publish. This form provides structure for consistency and transparency in reporting. For further information on Nature Research policies, see Authors & Referees and the Editorial Policy Checklist.

Statistics

For all statistical analyses, confirm that the following items are present in the figure legend, table legend, main text, or Methods section.

n/a	Confirmed	
☐	☒	The exact sample size (n) for each experimental group/condition, given as a discrete number and unit of measurement
☐	☒	A statement on whether measurements were taken from distinct samples or whether the same sample was measured repeatedly
☐	☒	The statistical test(s) used AND whether they are one- or two-sided<br>*Only common tests should be described solely by name; describe more complex techniques in the Methods section.*
☐	☒	A description of all covariates tested
☐	☒	A description of any assumptions or corrections, such as tests of normality and adjustment for multiple comparisons
☐	☒	A full description of the statistical parameters including central tendency (e.g. means) or other basic estimates (e.g. regression coefficient) AND variation (e.g. standard deviation) or associated estimates of uncertainty (e.g. confidence intervals)
☐	☒	For null hypothesis testing, the test statistic (e.g. F, t, r) with confidence intervals, effect sizes, degrees of freedom and P value noted<br>*Give P values as exact values whenever suitable.*
☒	☐	For Bayesian analysis, information on the choice of priors and Markov chain Monte Carlo settings
☒	☐	For hierarchical and complex designs, identification of the appropriate level for tests and full reporting of outcomes
☒	☐	Estimates of effect sizes (e.g. Cohen's d, Pearson's r), indicating how they were calculated

Our web collection on statistics for biologists contains articles on many of the points above.

Software and code

Policy information about availability of computer code

Data collection	N/a
Data analysis	N/a

For manuscripts utilizing custom algorithms or software that are central to the research but not yet described in published literature, software must be made available to editors/reviewers. We strongly encourage code deposition in a community repository (e.g. GitHub). See the Nature Research guidelines for submitting code & software for further information.

Data

Policy information about availability of data

All manuscripts must include a data availability statement. This statement should provide the following information, where applicable:

- Accession codes, unique identifiers, or web links for publicly available datasets
- A list of figures that have associated raw data
- A description of any restrictions on data availability

The authors declare that all data supporting the findings of this study are available upon request from the authors. The artefacts and faunal remains from the Fa-Hien Lena excavations are curated at the Department of Archaeology, Government of Sri Lanka, under the site code BYP and the suffixes 10, 11, and 12 (denoting the year of excavation). Some materials remained housed at the Max Planck Institute for the Science of Human History to be returned to the Department of Archaeology, Government of Sri Lanka by the end of 2019. All of the data reported in the paper are presented in the main text or in the Supplementary Notes, Tables, and Figures.

Field-specific reporting

Please select the one below that is the best fit for your research. If you are not sure, read the appropriate sections before making your selection.

☐ Life sciences ☒ Behavioural & social sciences ☐ Ecological, evolutionary & environmental sciences

For a reference copy of the document with all sections, see nature.com/documents/nr-reporting-summary-flat.pdf

Behavioural & social sciences study design

All studies must disclose on these points even when the disclosure is negative.

Study description

We apply new chronometric, geoarchaeological, taphonomic, archaeozoological, and artifactual analyses to the earliest dated archaeological site in Sri Lanka, Fa-Hien Lena, previously dated to 38,000 years ago. All forms of analyses involve numerical data. Some involve statistical evaluation and methodologies.

Research sample

The site of Fa-Hien Lena and its sedimentary, faunal, bone tool, lithic tool remains.

Sampling strategy

This paper presents the results of the analyses of materials from the 2009 to 2012 excavations. We added to existing radiocarbon dates and present a revised stratigraphy for the site. Together with dates previously published, which we calibrated using OxCal 4.3, we present a total of 30 radiocarbon dates (Supplementary Tables 1-4) that are now available for Fa-Hien Lena, enabling detailed phasing for the site.

For geoarchaeological analysis, a set of undisturbed sediment samples were collected from the excavated profile in clear polyurethane boxes. Sample boxes were labelled, photographed and plotted on the profile drawing before removal from the profile.

We analyzed faunal and bone tool remains recovered from the 2009 and 2012 excavations of Fa-Hien Lena. All bone fragments from sedimentary contexts with secure radiocarbon dates (including those from deposits sandwiched by dated layers) were included in the analysis.

The technological study of the lithic assemblages of Fa-Hien Lena was carried out following the chaîne opératoire concept, a methodological framework that defines the reconstruction of the various processes of flake production from the procurement of raw materials, through the phases of manufacture and utilization until the final discard. The chaîne opératoire concept provides systematic sequences of the flaking activities in which is possible to determine the temporal phase and the position of the artifact produced.The lithic material is composed of 5,070 items (Supplementary Tables 43-44). The predominant raw material is quartz which is abundantly available in the streams nearby.

Data collection

This paper presents the results of the analyses of materials from the 2009 to 2012 excavations. We added to existing radiocarbon dates and present a revised stratigraphy for the site. Together with dates previously published, which we calibrated using OxCal 4.3, we present a total of 30 radiocarbon dates (Supplementary Tables 1-4) that are now available for Fa-Hien Lena, enabling detailed phasing for the site.

For geoarchaeology, sample processing, at the Thin Section Micromorphology Laboratory, University of Stirling included air-drying and impregnation with polyester (polylite) resin (http://www.thin.stir.ac.uk/). c. 30 µm thick, covered, large format thin sections (7.5×11 cm) were manufactured from the hardened impregnated blocks (sample code FH). Thin sections were observed with a polarising microscope at magnifications of ×12.5 to ×400, using plain polarised (PPL), cross-polarised (XPL) and oblique incident light (OIL). The relative abundance of sediment components was estimated using standard semi-quantitative estimation charts.

All zooarchaeological specimens, including diaphyses and rib shafts, were sorted, counted, and measured (length, width, and thickness) using a digital caliper (Mitutoyo 500-463). Identified specimens were recorded in detail using codes for anatomic zones (e.g., Supplementary Figure 5) that would allow the description of bone preservation/fragmentation patterns. Diagnostic dental and skeletal elements were identified to the highest possible taxonomic level using vertebrate comparative collections from the Laboratory of Comparative Anatomy of the Muséum national d'Histoire naturelle (MNHN) in Paris and photographs from the mammalian collections of the Field Museum of Natural History and American Museum of Natural History. Following von den Driesch52, individual dental specimens and specific anatomical feature of diagnostic skeletal elements were measured to differentiate between closely related taxa. The naming of identified taxa follows the nomenclature for mammalian species by Wilson and Reeder53. The taxa identified in the sites were assigned to size class based on live weight (modified from 54,55): (a) micromammals: 100g to 1 kg, (b) small mammals: 1 kg to 25 kg, (c) large mammals class 1: 25 kg to 200 kg, (d) large mammals class 2: 200 kg to 1000 kg, (e) large mammals class 3: >1000 kg.

All fragments were examined for natural, animal, and anthropic modifications, including weathering, abrasion, burning, staining, and butchery marks32. Bone surface modifications were recorded/observed using an Olympus BX53 light microscope and a Keyence VHX-6000 digital microscope. Burnt bone fragments were identified based on color. They were distinguished into different classes based on the degree of burning56: (1) partially burnt, (2) charred (blackened), and (3) calcined (partial and complete calcination). Burnt bones were quantified by determining the percentage of the total bone fragments that comprises burnt specimens.

The technological study of the lithic assemblages of Fa-Hien Lena was carried out following the chaîne opératoire concept, a methodological framework that defines the reconstruction of the various processes of flake production from the procurement of raw materials, through the phases of manufacture and utilization until the final discard. The chaîne opératoire concept provides systematic sequences of the flaking activities in which is possible to determine the temporal phase and the position of the artifact produced.

For the analysis of osseous artifacts, the materials were examined using a Zeiss Stemi 508 stereomicroscope fitted with an AxioCam 105 camera. Taphonomic and anthropogenic alterations were identified based on published works58, 71-78 and mapped onto photographs

	taken with a Canon digital SLR camera.
Timing	2009-2018
Data exclusions	N/a
Non-participation	N/a
Randomization	N/a

Reporting for specific materials, systems and methods

We require information from authors about some types of materials, experimental systems and methods used in many studies. Here, indicate whether each material, system or method listed is relevant to your study. If you are not sure if a list item applies to your research, read the appropriate section before selecting a response.

Materials & experimental systems

n/a	Involved in the study
☒	☐ Antibodies
☒	☐ Eukaryotic cell lines
☒	☐ Palaeontology
☒	☐ Animals and other organisms
☒	☐ Human research participants
☒	☐ Clinical data

Methods

n/a	Involved in the study
☒	☐ ChIP-seq
☒	☐ Flow cytometry
☒	☐ MRI-based neuroimaging
